# Acute and chronic exposure to air pollution in relation with incidence, prevalence, severity and mortality of COVID-19: a rapid systematic review

**DOI:** 10.1186/s12940-021-00714-1

**Published:** 2021-04-10

**Authors:** Patrick D. M. C. Katoto, Amanda S. Brand, Buket Bakan, Paul Musa Obadia, Carsi Kuhangana, Tony Kayembe-Kitenge, Joseph Pyana Kitenge, Celestin Banza Lubaba Nkulu, Jeroen Vanoirbeek, Tim S. Nawrot, Peter Hoet, Benoit Nemery

**Affiliations:** 1 Department of Medicine and Centre for Infectious Diseases, Faculty of Medicine and Health Sciences, Stellenbosch University, Francie van Zijl Drive, Tygerberg, Cape Town, 7505 South Africa; 2 Department of Internal Medicine, Division of Respiratory Medicine & Centre for Global Health and Tropical Diseases, Catholic University of Bukavu, Bukavu, Democratic Republic of the Congo; 3 Centre for Evidence-Based Health Care, Division of Epidemiology and Biostatistics, Department of Global Health, Stellenbosch University, Cape Town, South Africa; 4 Department of Molecular Biology and Genetics, Faculty of Science, Ataturk University, 25240 Erzurum, Turkey; 5 Centre for Environment and Health, Department of Public Health and Primary Care, KU Leuven, Herestraat 49 (O&N 706), B-3000 Leuven, Belgium; 6 Unit of Toxicology and Environment, School of Public Health, University of Lubumbashi, Lubumbashi, Democratic Republic of Congo; 7 Department of Public Health, Faculty of Medicine and Public Health, University of Kolwezi, Kolwezi, Democratic Republic of the Congo; 8 Occupational Medicine and Environmental Health, Department of Public Health, Faculty of Medicine, University of Lubumbashi, Lubumbashi, Democratic Republic of the Congo; 9 Centre of Environmental Health, University of Hasselt, Hasselt, Belgium

**Keywords:** Short-term, Long-term air pollution, SARS-CoV-2, Burden, Susceptibility, Lethality

## Abstract

**Background:**

Air pollution is one of the world’s leading mortality risk factors contributing to seven million deaths annually. COVID-19 pandemic has claimed about one million deaths in less than a year. However, it is unclear whether exposure to acute and chronic air pollution influences the COVID-19 epidemiologic curve.

**Methods:**

We searched for relevant studies listed in six electronic databases between December 2019 and September 2020. We applied no language or publication status limits. Studies presented as original articles, studies that assessed risk, incidence, prevalence, or lethality of COVID-19 in relation with exposure to either short-term or long-term exposure to ambient air pollution were included. All patients regardless of age, sex and location diagnosed as having COVID-19 of any severity were taken into consideration. We synthesised results using harvest plots based on effect direction.

**Results:**

Included studies were cross-sectional (*n* = 10), retrospective cohorts (*n* = 9), ecological (*n* = 6 of which two were time-series) and hypothesis (n = 1). Of these studies, 52 and 48% assessed the effect of short-term and long-term pollutant exposure, respectively and one evaluated both. Pollutants mostly studied were PM_2.5_ (64%), NO_2_ (50%), PM_10_ (43%) and O_3_ (29%) for acute effects and PM_2.5_ (85%), NO_2_ (39%) and O_3_ (23%) then PM_10_ (15%) for chronic effects. Most assessed COVID-19 outcomes were incidence and mortality rate. Acutely, pollutants independently associated with COVID-19 incidence and mortality were first PM_2.5_ then PM_10_, NO_2_ and O_3_ (only for incident cases). Chronically, similar relationships were found for PM_2.5_ and NO_2_. High overall risk of bias judgments (86 and 39% in short-term and long-term exposure studies, respectively) was predominantly due to a failure to adjust aggregated data for important confounders, and to a lesser extent because of a lack of comparative analysis.

**Conclusion:**

The body of evidence indicates that both acute and chronic exposure to air pollution can affect COVID-19 epidemiology. The evidence is unclear for acute exposure due to a higher level of bias in existing studies as compared to moderate evidence with chronic exposure. Public health interventions that help minimize anthropogenic pollutant source and socio-economic injustice/disparities may reduce the planetary threat posed by both COVID-19 and air pollution pandemics.

**Supplementary Information:**

The online version contains supplementary material available at 10.1186/s12940-021-00714-1.

## Introduction

During the 2003 SARS outbreak in China, SARS patients from regions with high air pollution were twice as likely to die from SARS compared to those from regions with low air pollution, for both acute and chronic exposure to pollutants [1]. The World Health Organization (WHO) has announced a new SARS (SARS-CoV-2), responsible for the coronavirus disease-19 (COVID-19) as a global pandemic. According to WHO, 62.844.837 total cases and 1.465.144 deaths had been confirmed by 01/12/2020 [2]. Ambient air pollution constitutes a serious risk factor not only for the occurrence of respiratory infections, but also for the development of reduced pulmonary function and/or aggravation of existing pulmonary disease and is responsible for about four million deaths each year [3]. Exposure to pollutants can impair immune responses and affect host immunity from respiratory virus infections mainly in people already at risk of developing morbidity after viral respiratory infections [4, 5]. A recent meta-analysis [6] of 27,670 COVID-19 patients showed that the underlying morbidities associated with the highest likelihood of deaths consisted of cardiovascular diseases, immune and metabolic disorders, respiratory diseases, cerebrovascular diseases and cancer, i.e. conditions that are also known to be independently associated with exposure to pollutants [3, 7, 8]. Recently, it has been observed that in non-COVID-19 patients short-term exposure to particulate and gaseous air pollution prior to hospital admission is an important – modifiable – risk factor that prolongs the duration of ventilation [9]. However, studies reporting on the influence of pre-admission exposure to ambient air pollution on the duration of mechanical ventilation in COVID-19 patients admitted to the intensive care unit (ICU) are rare.

Inhalation of elevated concentrations of air pollution results in inflammation of mucus membranes in the pulmonary tract and is a factor that could influence the process and severity of SARSCov2-infection. Mechanistically, SARS-CoV-2 targets cells through the viral structural spike (S) protein that binds to the angiotensin-converting enzyme 2 (ACE2) receptor. Bronchial epithelial cells, type I and type II alveolar pneumocytes, and capillary endothelial cells become infected, and an inflammatory response ensues [10]. Increased viral load reduces the expression of ACE2 and contributes to an aggressive reaction by multiple cytokines and chemokines, and to a lowering of innate immunity by adaptive and regulatory immune cells [11]. CD4+ T cells have been shown to allow antibody development and a healthy immune response. During SARS-CoV-2 infection, lymphopenia develops early and is prognostic, possibly correlated with a decrease of CD4+ and certain CD8+ T cells. T cells are selectively killed by the immune system, while there is a risk of direct viral invasion of T cells [12]. Bioinformatics review of the DNA sequence encoding SARS-CoV-2 cell identified nine consensual patterns for the aryl hydrocarbon receptor and thus supported the hypothesis that pollution-induced over-expression of ACE-2 in human airways can favor SARS-CoV-2 infection [13]. Ultimately, a “double-hit hypothesis” was recently suggested: prolonged exposure to PM2.5 induces overexpression of the alveolar ACE-2 receptor. This can raise viral loads in patients exposed to pollutants and in exchange, deplete the ACE-2 receptors and damage the host defense. High atmospheric NO2 may provide a second hit causing a severe form of SARS-CoV-2 in ACE-2 depleted lungs resulting in a worse outcomes [14]. Moreover, air pollution may not only promote a longer presence of viral particles (conflicting evidence) in the air and contribute to the spread of SARS-CoV-2, but may in the case of pre-exposure to pollutants, sustain an inflammatory storm triggered by SARS-CoV-2 [15]. Interleukins (IL), interferons (IFN), tumor necrosis factor (TNF), colony stimulating factors (CSF), the chemokine family, growth factors (GF) are the major cytokines concerned [16]. Cytokine storm is a primary cause of acute respiratory distress syndrome [16] that is known to be associated with air pollutants in critically ill patients [17, 18]. The variations in reaction are presumably related to the degree of viral load and host-related factors [16, 19]. Persistent immune activation in predisposed individuals, such as elderly adults and those at cardiovascular risk, will contribute to hemophagocytosis-like syndrome, with excessive amplification of cytokine development contributing to multi-organ failure and death [12].

We performed a rapid systematic review to summarize the existing – and still somewhat controversial [20] – information on the subject. Compared to already published reviews, we took great care to expanding the search terms, assessing the quality of studies, documenting both acute and chronic exposure, summarizing findings by epidemiological and clinical COVID-19 outcomes and by ascertaining the degree of certainty for each included study.

## Methods

This review is reported according to Preferred Reporting Items for Systematic reviews and Meta-Analysis protocol (PRISMA-P) guidelines [21, 22].

### Search strategy and data extraction

The search strategy has been applied using online databases (PubMed/MEDLINE, Google Scholar, Embase, Web of Science, WHO COVID-19 database, Cochrane Library) from December 2019 to September 2020. Preprint papers indexed in Medline (MedRxiv) were included and were considered as grey literature until peer reviewed versions were available. No language restriction was applied. The literature search technique was developed using the headings of the medical subject headings (MeSH), Boolean (AND/OR) operator. The search terms used included: (1): “air pollution”, “outdoor air pollution”, “ultra fine particles”, “fine particles”, “coarse particles”, “traffic related pollutants”, “traffi c”, “diesel”, “elemental carbon”, “black carbon”, “particulate matters”, “nitrogen dioxide”, “carbon monoxide”, “nitrogen dioxide”, “nitrogen oxides”, “ozone”, “sulfur dioxide”, “sulfur oxides”, “O_3_”, “SO_2_”, “SOx”, “NO_2_”, “NOx”, “PM”, “particulate matter”, “air pollution”, “ultrafine particles”, “PM_10_”, “PM_2·5_”, “PM_1_”, “bioaerosols in PM”, “bacteria in PM”, “endotoxin in PM”, “fungi and pollens in PM”, “trace elements in PM”, “secondary inorganic species in PM”, “polycyclic aromatic hydrocarbon in PM”, “inorganic mineral dust in PM”, “elemental carbon in PM”, “organic carbon in PM”, and “black carbon in PM”, volatile organic chemical or volatile organic compound”, “VOCS”; (2): “Wuhan coronavirus” OR “COVID-19″ OR “novel coronavirus” OR “2019-nCoV” OR “coronavirus disease” OR “SARS-CoV-2″ OR “SARS-2″ OR “severe acute respiratory syndrome coronavirus 2” and (3) “admission” OR “outcome” OR “case fatality rate” OR “CFR” OR “mortality” OR “lethality” OR “prevalence” OR “incidence” OR “prevalence of asymptomatic, mild, moderate and severe cases” OR “number admitted to specialized units or intensive care units” OR “number of infected patients”. The final search included (1) AND (2) AND (3). Searching results was independently evaluated by two different reviewers (PDMK and BB).

### Selection and data collection process

Full texts for the eligible titles and/or abstracts, including those where there was uncertainty, were obtained for further assessment on whether to include in the study or not [23]. Disagreements between authors were resolved through discussion and, when needed, there was arbitration by a third reviewer (AB). Reasons for excluding articles were recorded. For studies appearing in more than one published article, we considered the most recent one, and with the largest sample size. For surveys appearing in one article with multiple surveys conducted at different time points, we treated each survey as a separate study. For multi-national studies, estimates at the country level were preferred. Data was extracted using a standardized data extraction form. From the studies included, two reviewers (PDMK and BB) independently extracted data using the predefined standardized extraction form. Data extracted comprised information about the study ID, study description, source, type and length of exposure, COVID-19 outcomes investigated, main findings and conclusion.

### Handling of preprint publications

Preprint papers indexed in Medline (MedRxiv) were included and were considered as part of grey literature at inclusion and data were interpreted cautiously. However, after the peer-reviewed process of our article, we considered and replaced the preprint versions by the peer-reviewed articles, if available. When necessary, we cited the two versions for methodological purposes.

### Inclusion and exclusion criteria

Studies presented as original articles, studies that assessed risk, incidence, prevalence, or lethality from COVID-19 in relation with both exposure to short-term and long-term exposure to ambient air pollution were included.

#### Types of studies

Observational studies (including ecological, cross-sectional, case–control, and cohort designs) were included. We also included a summary of already published reviews.

#### Participants

All patients regardless of age, sex and location diagnosed as having COVID-19 of any severity.

#### Intervention(s)/exposure(s)

Length and level of exposure to air pollutants.

#### Outcome

Epidemiological data (prevalence, incidence, absolute number of cases) and clinical data (case fatality rate, mortality rate, absolute death number) of COVID-19.

#### Settings

Worldwide, either hospital-based or register-based.

#### Exclusion criteria

Studies not performed in humans or qualitative studies, and studies that lack relevant data needed to compute the outcome for COVID-19 or to describe the type of air pollution (level or length of exposure) were excluded. Experimental studies, letters, comments, editorials, case reports, and case series were not included.

### Quality assessment and risk of bias in individual studies

As recently reported by a panel of experts [24, 25]; currents risk of bias (RoB) tools tend to dilute the quality of study assessing adverse effects of environmental exposures. This occurs by incorrectly excluding studies contributing to misleading the quality of the body of evidence. To determine the overall RoB of the studies included in this review, we built on the approach described by Lee et al. 2020 [26] and considered each study individually in the summary of evidence. Briefly, we assessed three key domains of interest in observational studies: (1) objective assessment of outcomes, (2) adjustment for confounding using baseline covariates as well as exposure level and (3) whether a control/dose-response comparator was used for comparative analysis. For the objective assessment of outcomes, we judged studies using an objective measure of outcome assessment (such as physician diagnosis, clinical information, or ICD codes) as low risk for detection bias. Studies using subjective measures, such as participant recall, were judged as high risk for this domain. If it was not clear how outcomes were assessed, we judged such studies at unclear risk of detection bias.

Adjustment for confounding was assessed based on the covariates used to adjust results. Studies which adjusted for age, in addition to two other relevant covariates, or which reported on individual levels of exposure were judged at low risk for this domain; studies which did not adjust results for at least age and reported aggregated levels of exposure were judged as high risk. Those studies that adjusted for age, but no other covariates, and either reported aggregated exposure, or did not clearly state whether exposure was reported at individual or aggregated level, were judged as unclear risk. To arrive at an overall risk of bias judgment, the three key domains were considered together. If a study was at low risk for all key domains, it was judged as having a low overall risk of bias. If any of the domains were unclear and the others low, overall risk of bias would be judged unclear. If any of the domains were high; and the others judged low, unclear, or high; overall risk of bias would be high for the study.

### Data management

Based on the inclusion and exclusion criteria, a tool was developed a priori to guide the screening and selection process. The tool was piloted and revised before beginning data extraction. The search results were uploaded to Zotero software to remove duplicates.

### Evidence synthesis by harvest plots

As a result of the diverse measures of associations and analytical approaches used in the included studies, the variation in the measurement and reporting of the same outcomes (e.g. diagnosis of COVID-19 or reporting of case fatality rate (CFR) in different countries), incomplete reporting of necessary data (e.g. variances) and the urgent need for evidence, we did not pool results in a traditional meta-analysis. Instead, we synthesized effects of different pollutant exposures on different outcomes using harvest plots as described by Ogilvie and colleagues [27] and used by a Cochrane systematic review by Durão et al., 2020 [28].

The method does not account for the relative weight of studies, as it does not take variance into account, but it does provide insight into the direction of the overall results based on a vote-counting approach, and it can provide a robust approximation of certainty. Point estimates of association were used to determine the direction of effect for each study, and 95% confidence intervals (CIs) were used to determine the certainty of the direction; when the 95% CIs included the line of no association, the effect was considered as uncertain. Where 95% CI were not available, *p*-values were used to make a judgment about certainty. Though we acknowledge the limitations of this measure for discerning ‘precise’ and ‘imprecise’ associations, this approach enabled us to make robust decisions about whether reported associations were convincing or not. In these cases, p-values above 0.05 were considered to indicate an uncertain association.

The determination of overall direction of association and certainty was based on where most studies were located in the harvest plot, whether studies provided contradictory evidence and on the quality of the individual studies. If most studies were clustered in a particular direction, certainty was determined by the number of studies falling in the ‘uncertain’ categories. If studies reported effects in different directions, their numbers in either direction, overall risk of bias, as well as the certainty of the evidence presented were used to determine an overall direction. If studies were equally distributed in either direction and of equal certainty and quality, the overall effect was deemed to be very uncertain. As quantitative syntheses were not conducted, the GRADE approach [29] for assessing the certainty of the evidence was not followed formally, but the domains used in this approach were considered when making certainty statements.

### Other evidence considered

**Fig. 1:**
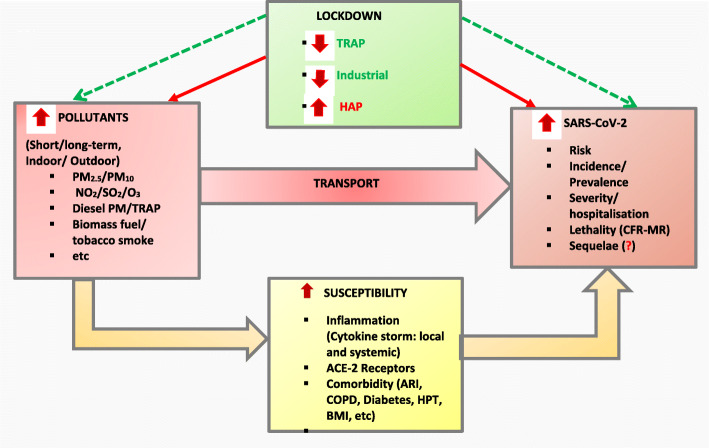
Interplay of Air pollution, Lockdown and SARS-CoV-2: An Epidemiological View. Model built following synthesis of current litterature [3, 13, 15, 30–51] . The airborne nature of SARS-CoV-2 transmission might be facilitated by air pollutants. Indirectly pollutant can increase host susceptibility to SARS-CoV-2 by directly induce respiratory epithelium/ endothelium lesions. Further, pollutants trigger oxidative stress, increase ACE-Receptors, and are independently associated with the risk, severity, and mortality for cardiorespiratory and metabolic diseases (COPD, tuberculosis, ARI, HTP, high BMI, diabetes, etc.). Patently, SARS-CoV-2 manifestation is linked to cytokine storm liberation, it binds to ACE-2 Receptors to penetrate host cell membrane and is more severe among people with the above evoked cardiorespiratory and metabolic conditions. In addition, pollutant can sustain cytokine storm triggered by SARS-CoV-2. Consequently, exposure to high level of pollutants potentiates SARS-CoV-2 effect resulting in increased risk, incidence, severity, and lethality with uncertain level of evidence related multiorgan sequelae. On the other hand, COVID-19 pandemic has resulted into a lockdown which has clearly improved the level of anthropogenic pollutants. Not such benefice is expected for household burning solid biomass fuel for domestic energy or containing a smoker as strict lockdown has resulted on the increased exposure-time. Abbreviations: SARS-CoV-2: severe acute respiratory syndrome coronavirus; PM_2.5_ (or _10_): particulate matter of less than 2.5 (or 10) micrometers in diameter, NO_2_: nitrogen dioxide; O_3_: ozone; SO_2_: sulfur dioxide; TRAP: traffic related air pollution; HAP: household air pollution; ACE-2: angiotensin-converting enzyme 2 ARI: acute respiratory infection; COPD: chronic obstructive pulmonary diseases; HPT: hypertension; BMI: body mass index; CFR: case fatality rate; MR: mortality rate

As recently suggested by Steenland and colleagues [25], we did not only consider human primary studies to assess the evidence but we also considered triangulation and integration of animal and mechanistic data and summary of reviews to have a broad approach by constructing an epidemiological pathway of the association between exposure to pollutants, effect of lockdown and SARS-CoV-2 infection (Fig. 1).

## Results

### Search findings and characteristics of included studies

**Fig. 2:**
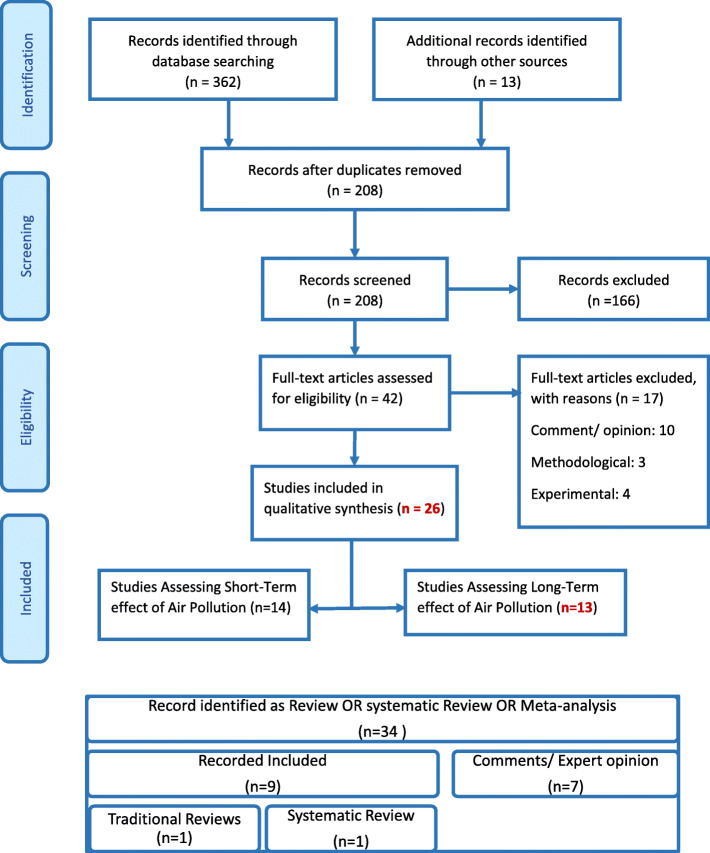
PRISMA Flow Diagram. Note: One study has assessed both short-term and long-term air pollution

Overall, we have included 26 primary studies and summarized nine reviews (Fig. 2). eTable 1 displays the summary of reviews and eTable 2 shows the characteristic of primary studies. Primary studies were cross-sectionals (*n* = 10), retrospective cohorts (*n* = 9), ecological (*n* = 6 of which two were time-series) and hypothesis (*n* = 1). Eleven originated from Europe (Italy: 9), eight from Asia (China: 6), five from the USA and two from Latin America. About 52 and 48% of studies assessed the effect of short-term and long-term exposure to pollutants, respectively while one study evaluated both concomitantly. Pollutants mostly studied for acute effects included PM_2.5_ (64%), NO_2_ (50%), PM_10_ (43%) and O_3_ (29%). Equally, for chronic effect it was mostly PM_2.5_ (85%), NO_2_ (39%) and O_3_ (23%) then PM_10_ (15%).

### Quality of included studies

**Fig. 3:**
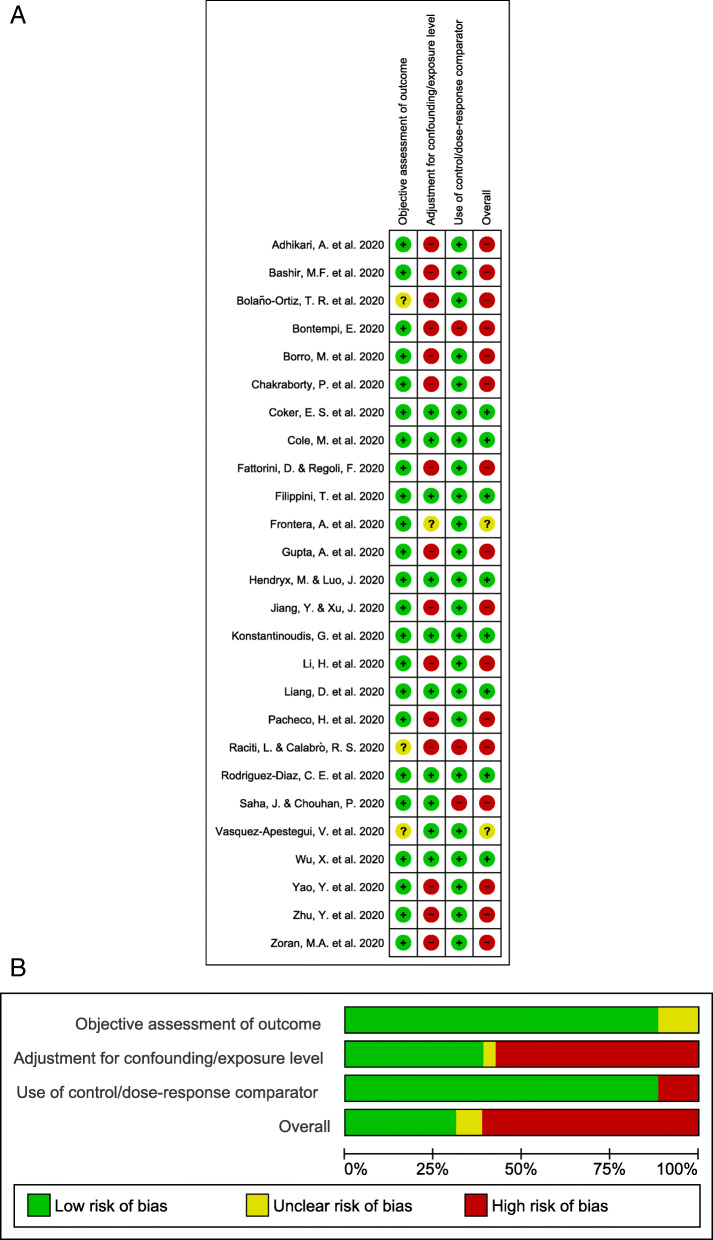
Risk of bias. Summary of authors’ judgments on each domain for each included study (Panel **a**) and as percentages across included studies (Panel **b**). About one quarter of the studies had a low risk of bias

Of the 26 studies included in the review, 16 had a high overall risk of bias when assessing the three key domains (Fig. 3, eTables 3 and 4). One study [52] reported on short-term and long-term exposures and was assessed for both. Twelve of the 14 (86%) studies reporting on short-term exposure to pollutants, and five of the 13 reporting on long-term exposure (39%), were at a high overall risk of bias. High overall risk of bias judgments were predominantly due to a failure to adjust aggregated data for age and other important covariates as confounders, and to a lesser extent as a result of a lack of comparative analysis in two short-term studies [30, 53] and in one long-term study [54]. Of the remaining studies reporting on short-term exposure to pollutants, one was at low overall risk of bias [55] and the other [14] at unclear overall risk of bias; due to having adjusted aggregated data for age only. Seven of the remaining studies reporting on long-term exposure were at low overall risk of bias. One study [56] was at unclear overall risk of bias as it was not clear where authors had obtained COVID-19 outcome data, or how these outcomes were determined.

### Overview of published reviews

Of the nine included reviews, seven were written as comment or expert opinion, one was a traditional review, and one was a systematic review including 15 studies. None of the reviews quantitatively assessed the quality of included studies, nor did they consider an adapted approach for GRADEing the summary of findings to support making recommendations and to determine the strength of their recommendations (eTable 1).

### Evidence synthesis by harvest plots

#### Effects of short-term air pollution on COVID-19-related deaths

**Fig. 4:**
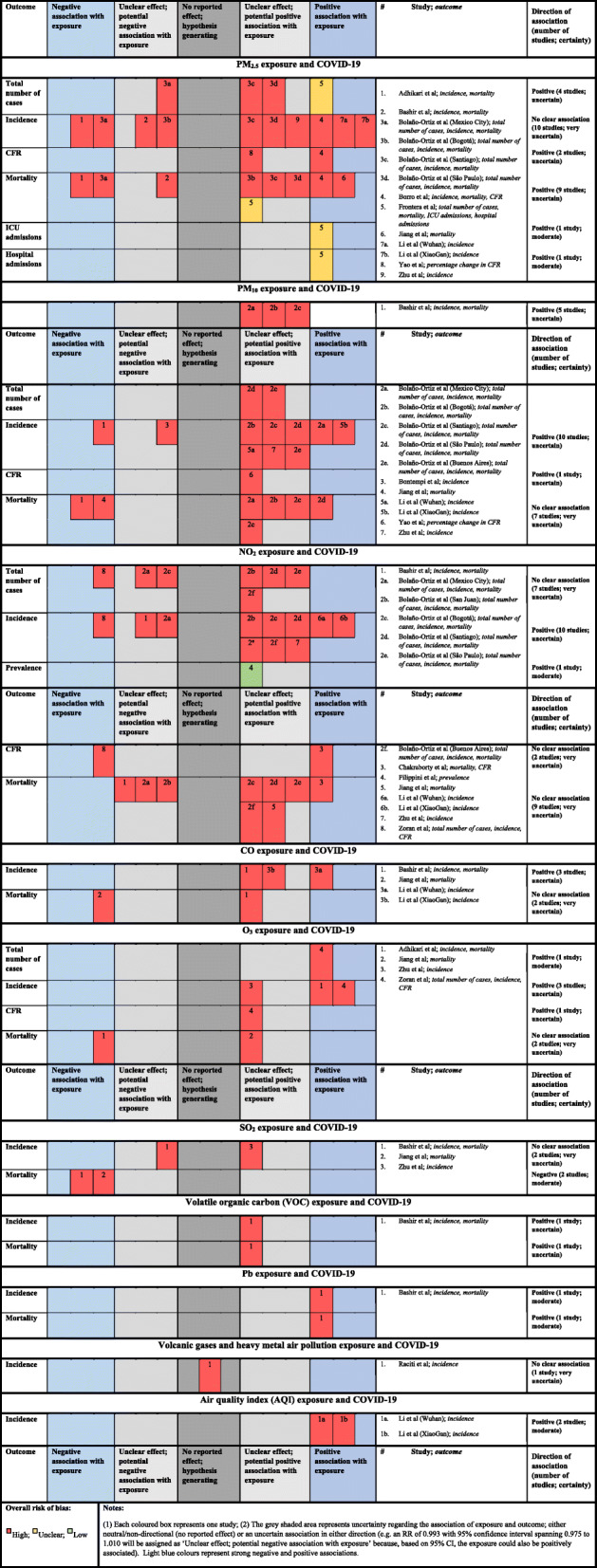
Harvest plots displaying level of evidence between short-term exposure to air pollution and risk, severity, incidence, and lethality for COVID-19 Pandemic

Table 1 displays the associations between short-term exposure to various pollutants and COVID-19 outcomes; Fig. 4 displays the related quality of evidence. Overall, short-term exposure to PM_2.5_ has been significantly associated with all COVID-19-death related outcomes. For instance, a 10 μg/m^3^ increase in PM_2.5_ was associated with a 0.24% (0.01–0.48%) increase in case fatality rate (CFR) in 49 Chinese cities [52], and an incidence rate ratio (IRR) of 1.079 (95%CI: 1.071–1.086) for total deaths in Wuhan (China) [64]. However, a study in New York (USA) [60] failed to demonstrate a significant association between short-term exposure to PM_2.5_ and increased risk of death (PM_2.5__lag0–21: IRR: 0.8912, 95%CI: (0.7966–0.9971) and suggested to adjust for other risk factors. Two studies reporting associations of short-term exposure to PM_2.5_ and CFR were at high overall risk of bias and reported positive associations of differing precision, leading to an uncertain positive association. The evidence for the association of short-term PM_2.5_ exposure with mortality was contradictory, with three studies at high overall risk of bias showing associations in a negative direction, while five studies at high overall risk of bias and one study at unclear overall risk of bias indicated positive associations of varying precision. In sum, there is a potential positive association between short-term PM_2.5_ exposure and mortality, but the evidence is uncertain.

**Table 1:** Association between short-term exposure to air pollution and risk, severity, incidence, and lethality for COVID-19 Pandemic

Study ID	Study Description	Outcomes	Main findings	Conclusion
Yao Y et al. [52], June 2020	*Associations between PM and COVID-19 CFR *49 Chinese cities, spatial analysis	CFR	Pollutants (10 μg/m^3^ increase in and concentrations)- COVID-19 CFR increased by: *Epidemic period: • PM_2.5_: 0.24% (0.01–0.48%) and • PM_10_: 0.26% (0.00–0.51%), respectively.	PM distribution and its association with COVID-19 CFR suggests that exposure to such may affect COVID-19 prognosis.
Frontera A et al. [14], August 2020	*Relationship between air PM_2.5_ and NO_2_ and COVID-19, Italian regions.	Transmission, number of patients, severity of presentation and number of deaths.	*Correlations between mean PM_2.5_: • Total number cases: r = 0.64; *p* = 0.0074, • ICU admissions per day: r = 0.65; *p* = 0.0051, • Deaths: r = 0.62; *p* = 0.032 • Hospitalized cases: r = 0.62; *p* = 0.0089	*Highest cases, more severe cases and two-fold mortality of COVID-19 in the most polluted regions
Li H [57] et al., August 2020	Retrospective study, correlation between COVID-19 incidence and AQI, Wuhan and XiaoGan between January 26th to February 29th in 2020	Incidence	* Pollutants-COVID-19 Incidence (Wuhan vs XiaoGan: R2): • PM_2.5_: 0.174 vs 0.23 • PM_10_: 0.105 vs 0.158 • NO_2_: 0.329 vs 0.158 • CO: 0.203 vs 0.022 *AQI-COVID-19 Incidence (Wuhan vs XiaoGan: R^2^): 0.13, *p* < 0.05 vs 0.223, *p* < 0.01).	AQI, PM_2.5_, NO_2_, and temperature are four variables that could promote the sustained transmission of COVID-19.
Zoran M et al. [58], October 2020	Time series of daily average inhalable gaseous O_3_ and NO_2_, in Milan, Lombardy in Italy, January–April 2020	Transmission and lethality	O_3_ vs NO_2_ (January–April 2020)-COVID-19: • Total number: r = 0.64 **vs − 0.55** • Daily New positive: r = 0.50** vs − 0.35** • Total Deaths cases: r = 0.69 vs − 0.58**	* O_3_ can acts as a COVID-19 virus incubator. *Estimates can be attributed to airborne bioaerosols distribution.
Zhu Y et al. [59], Jully 2020	Daily confirmed cases, air pollution concentration and meteorological variables in 120 cities were obtained from January 23, 2020 to February 29, 2020 in China.	Incidence	*10-μg/m^3^ increase (lag0–14) associated with increase in the daily counts of confirmed cases: • PM_2.5_: 2.24% (95% CI: 1.02 to 3.46), • PM_10_: 1.76% (95% CI: 0.89 to 2.63), • NO_2_:6.94% (95% CI: 2.38 to 11.51), • O_3_: 4.76% (95% CI: 1.99 to 7.52) *10-μg/m^3^ increase (lag0–14) associated with a decrease in COVID-19 confirmed cases. SO_2_: 7.79%: (95% CI: − 14.57 to − 1.01)	Significant relationship between air pollution and COVID-19 infection, which could partially explain the effect of national lockdown and provide implications for the control and prevention of this novel disease.
Adhikari A et al. [60], Jun 2020	Associations between O_3_, PM_2.5_, daily meteorological variables and COVID-19 in Queens county, New York during March–April 2020	Incidence and mortality	*Pollutants (lag 0–21)-New COVID-19 Cases • PM_2.5_: IRR: 0.6684 (0.6478–0.6896), *P* < 0.0001 • O_3_: IRR: 1.1051 (1.0747–1.1363), P < 0.0001 *Pollutants (lag 0–21)-New COVID-19 Deaths • PM_2.5_: IRR: 0.8912 (0.7966–0.9971), *P* < 0.0444 • O_3_: IRR: 0.8958 (0.8072–0.9941), *P* < 0.0382	Short-term exposures to O_3_-8h + other meteorological factors can influence COVID-19 transmission and initiation, but aggravation and mortality depend on other factors.
Chakraborty P et al. [61], July 2020	*Effects of COVID-19, on a large population persistently exposed to various pollutants in different parts of India. *Data, from online resources,	Fatality	*NO_2_ from vehicular emission and absolute number of COVID-19: • Deaths: r = 0.79, p < 0.05 • Case fatality rate: r = 0.74, *p* < 0.05. *Rise in NO_2_/ PM_2.5_ ratio increased the COVID-19 CFR by: 7.2%	Homeless, poverty-stricken, hawkers, roadside vendors, and others regularly exposed to vehicular exhaust, may be at a higher risk in the COVID-19 pandemic.
Bontempi E [30], July 2020	PM_10_ situation in Lombardy (from 10th February to March 27, 2020), several days before the sanitary emergency explosion; comparison: the situation of Piedmont, located near to the Lombardy	Incidence	Piedmont cities, presenting lower detected infections cases in comparison to Brescia and Bergamo in the investigated period, had most sever PM_10_ pollution events in comparison to Lombardy cities.	Not possible to conclude that COVID-19 diffusion mechanism also occurs through the air, by using PM10 as a carrier.
Bashir MF et al. [62], May 2020	Secondary published data from the Centers for Disease Control and the EPA (March–April 2020) to assess the relation between environmental pollution determinants and the COVID-19 outbreak in California.	Incidence, Mortality	*Pollutants-COVID-19 Cases • PM_10_: r = − 0.375** • PM_2.5_: r = − 0.453*** • SO_2_: r = − 0.426*** • CO: r = 0.083 • VOC: r = 0.054 • Pb: r = 0.178** • NO_2_: r = − 0.736*** *Pollutants-COVID-19 Deaths • PM_10_: r = − 0.350** • PM_2.5_: r = − 0.429*** • SO_2_: r = − 0.397** • CO: r = 0.123 • VOC: r = 0.038 • Pb: r = 0.174** • NO_2_: r = − 0.731***	Useful supplement to encourage regulatory bodies to promote changes in environmental policies as pollution source control can reduce the harmful effects of environmental pollutants
Bolaño-Ortiz TR et al. [63], July 2020	Correlation between air pollution indicators (PM_10_, PM_2.5_, and NO_2_: day 0–14 prior COVID-19 test) with the COVID-19 daily new cases and deaths in Latin America and the Caribbean region	Transmission and mortality	Spearman rank correlation tests: Mexico City (Mexico), PM_2.5_, PM_10_, NO_2_ • New Cases: − 0.214*, − 0.327**, − 0.206 • Total Cases: − 0.124, − 0.444***, − 0.446*** • Mortality: − 0.256**, − 0.395***, − 0.462*** San Juan (Puerto Rico), NO_2_: • New Cases: 0.367*** • Total Cases: 0.636*** • Mortality: − 0.194 Bogotá (Colombia), PM_2.5_, PM_10_, NO_2_ • New Cases: − 0.414***, − 0.150, 0.009 • Total Cases: PM_10_, NO_2_: − 0.438***, − 0.190 • Mortality: 0.050, 0.097, 0.182 Santiago (Chile), PM_2.5_, PM_10_, NO_2_ • New Cases: 0.466 ***, 0.351***, 0.547*** • Total Cases: 0.481***, 0.353***, 0.547*** • Mortality: 0.478***, 0.404 ***, 0.569 *** São Paulo (Brazil) PM_2.5_, PM_10_, NO_2_ • New Cases: 0.350***, 0.354***, 0.506*** • Total Cases: 0.261, 0.277, 0.337*** • Mortality: 0.203, 0.228*, 0.354*** Buenos Aires (Argentina). PM_10_, NO_2_ • New Cases: 0.414, 0.274 • Total Cases: 0.434***, 0.195 • Mortality: 0.157, 0.056	*COVID-19 infection rate correlation, in particular for the Gini index of each country (r = 0.51,*p* < 0.13), the urban poverty rate (r = − 077,*p* = 0.01) and the urban extreme poverty rate (r = 0.79, p = 0.01). *Income inequality and poverty levels in the cities analysed related to the spread of COVID-19 positive and negative, respectively.
Borro M et al. [13], August 2020	* PM_2.5_ and COVID-19 outcomes from 20 February-31 March 2020 in 110 Italian provinces *Bioinformatic analysis of the DNA sequence encoding the SARS-CoV-2 cell receptor ACE-2	Incidence, CFR, Mortality	*PM2.5 levels and COVID-19 • Incidence: r = 0.67, *p* < 0.0001) • Mortality rate: r = 0.65, p < 0.0001 • CFR: r = 0.7, p < 0.0001) *Bioinformatic analysis of the ACE-2 gene identified nine putative consensus motifs for the aryl hydrocarbon receptor.	*Confirm the supposed link between air pollution and the rate and outcome of SARS-CoV-2 infection *Support the hypothesis that pollution-induced over-expression of ACE-2 on human airways may favor SARS-CoV 2 infectivity
Raciti L et al. [53], Jun 2020	To assess the relationship between volcanic ash pollution and COVID-19 in Sicily, Italy	Incidence	Volcanic gases and heavy metals-related air pollution, combined to specific climatic conditions and regional topography, in favouring severe COVID-19 diffusion in Sicily	Clinical and epidemiological studies are needed to support the hypothesis
Jiang Y et al. [64], 2020 August	Retrospective study of ambient air pollutant concentrations (daily average), and meteorological variables data of Wuhan, Jan 25 and April 7, 2020 in relation to COVID-19	Death Number	*Pollutants-COVID-19 Deaths (RR, 95%CI, *p*-value): • PM_2.5_: 1.079, 1.071–1.086, < 0.01 • PM_10_: 0.952, 0.945–0.959, < 0.01 • SO_2_: 0.951, 0.919–0.984, < 0.01 • CO: 0.177, 0.131–0.24, < 0.01 • NO_2_: 1.002, 0.996–1.007, 0.55 • O_3__8h: 1.001, 0.998–1.003, 0.56	PM_2.5_ and diurnal temperature range are tightly associated with increased COVID-19 deaths.
Filippini T et al. [55], October 2020	Collection of NO_2_ tropospheric levels using satellite data available at the European Space Agency before the lockdown in association with COVID-19 at different time (March 8, 22 and April 5), in the 28 provinces of Lombardy, Veneto and Emilia-Romagna (Italy).	Prevalence rate	*Little association of NO_2_ levels with COVID-19 prevalence up to about 130 μmol/m2 *Positive association, evident at higher levels at each time point.	Notwithstanding the limitations of the use of aggregated data, these findings lend some support to the hypothesis that high levels of air pollution may favour the spread of the SARS-CoV-2 infection.

*Abbreviations*: *PM*_*2.5*_ *and*_*10*_ Particulate matter of diameter ≤ 2.5 and ≤ 10 μm respectively, *O*_*3*_ Ozone, *CO* Carbon monoxide, *SO*_*2*_ Sulfur dioxide, *NO*_*2*_ Nitrogen dioxide, *Pb* Lead, *CH*_*4*_ Methane. *ICU* Intensive care unit, CFR Case fatality rate, *AQI* Air quality index, *VOC* Volatile organic compounds, *IQR* Interquartile range, *ACE-2* Angiotensin-Converting Enzyme 2, *IRR* Incidence rate ration. *US EPA* United States Environmental Protection Agency

Acute exposure to PM_10_ has been associated with COVID-19 CFR in 120 cities in China [59] [CFR: 0.26% (0.00–0.51%) for 10 μg/m^3^ increase in PM10] and significantly correlated with mortality in Sicily (Italy) [62], Mexico City (Mexico), Santiago (Chili), with borderline correlation in Sao Paulo (Brazil) and no association in Bogota (Colombia) and Buenos Aires (Argentina) [63]. The association of short-term PM_10_ exposure and CFR is uncertain, with a single study at high overall risk of bias providing an unclear positive association. The overall association of short-term PM_10_ with mortality is very uncertain. All studies were at high overall risk of bias and provided conflicting evidence for the direction of association, with two studies indicating precise negative associations in contrast to one study providing a precise positive association and four studies showing imprecise, potentially positive associations.

Significant positive correlations between acute exposure to NO_2_ and COVID-19 related-deaths were reported in India [61], Santiago and Sao Paulo [63] while significant negative correlations were observed in an Italian region (Lombardy and Milan) [58], California [62] and Mexico [63]. However, a lack of significant correlations was observed in San Juan, Bogota, Buenos Aires [63] and in Wuhan [64]: RR (95%CI): 1.002, 0.996–1.007. The overall effect of short-term NO_2_ on CFR is very uncertain. Both studies were at high overall risk of bias and provided conflicting evidence for the direction of association. The evidence for the association of short-term NO_2_ exposure with mortality was contradictory, with three studies showing imprecise associations in a negative direction, while five studies indicated imprecise associations in a positive direction; a sixth study provided a precise positive association. All studies were at high overall risk of bias, and, hence, the evidence is very uncertain. A moderate but nonsignificant correlation was observed between short-term exposure to O_3_ and COVID-19 related deaths in Italy [58]. However, while a protective effect was reported in New York (O_3_: IRR: 1.1051 (1.0747–1.1363) [60], a null association was seen in Wuhan for very short term exposure (IRR (95%CI): 1.001, 0.998–1.003) [64]. The association of short-term O_3_ exposure with CFR is uncertain and based on a single study, also at high overall risk of bias, providing unclear results of a potentially positive association. The association of short-term O_3_ exposure with mortality was very uncertain, for the same reasons.

#### Effects of short-term air pollution on COVID-19 susceptibility

In most cases, short-term exposure to PM_2.5_ has been associated with COVID-19 incidence. Zhu [59] reported that a 10-μg/m^3^ increase (lag0–14) was associated with a 2.24% (95% CI: 1.02 to 3.46) increase in the daily counts of confirmed cases in 120 Chinese cities. Similar patterns were observed in Italian regions [13, 14], California (USA) [62] and in Latin American cities (Mexico, Santiago, Bogota, and Sao-Paulo) [63]. However, no such direction was observed in the city of New-York [lag 0–21-New COVID-19 Cases: IRR: 0.6684 (0.6478–0.6896)] [60]. In Italy, a significant relationship was demonstrated between exposure to short-term increases in PM_2.5_ and COVID-19 severity (hospitalization: r = 0.62; intensive care unit admission: r = 0.65) [14]. Most of the evidence, from studies at high overall risk of bias, indicates that short-term PM_2.5_ exposure is potentially positively associated with the total number of cases. One study at unclear overall risk of bias provides evidence of a precise positive association between PM_2.5_ and the outcome, but one study from Mexico City [63] provides conflicting evidence of an imprecise negative association. The overall effect, therefore, is an uncertain positive association. The evidence of the association of short-term PM_2.5_ exposure with COVID-19 incidence was contradictory, with four studies showing associations in a negative direction and six studies indicating associations in a positive direction. All studies were at high overall risk of bias, and the evidence is very uncertain. The association of short-term PM_2.5_ exposure with hospital admissions is positive and of moderate certainty, based on a single study at unclear overall risk of bias [14]. A single study at unclear overall risk of bias [14] reported on the association of short-term PM_2.5_ exposure and ICU admissions, providing a positive association of moderate certainty.

There was a huge disparity in correlation between acute exposure to PM_10_ and COVID-19 incidence. For example, a significant correlation was observed in the USA [62] but not in Italy [30]; in Mexico, Santiago and Sao-Paulo but not in Bogota [63] and not across Chinese cities [59]. All the evidence indicates that short-term PM_10_ exposure is potentially positively associated with the total number of cases, but all included studies provided unclear effects. The association of short-term PM_10_ with total cases is positive but uncertain. The evidence regarding short-term PM_10_ exposure and COVID-19 incidence was also contradictory, but most studies indicated an association in the positive direction. All studies were at high overall risk of bias, and most studies reported unclear associations; the resultant overall association is considered to be positive, but uncertain.

For every 10-μg/m^3^ increase (lag0–14) in NO_2_ concentration, the daily counts of confirmed COVID-19 cases also increased by 6.94% (95% CI: 2.38 to 11.51) across 120 Chinese cities [59]. Moreover, similar observations were described in studies focused on Wuhan and XiaoGan (China) [57], in San Juan and in Sao Paulo [63]. However, negative correlations were observed in Italy (Lombardy and Milan) [58], California [62] and in Mexico (for total cases) while no such correlations were reported in Mexico (New cases), Bogota and Buenos Aires [63]. Furthermore, little association of NO_2_ levels with COVID-19 prevalence was described across 28 provinces in Italy [55]. The overall association of short-term NO_2_ with total cases is very uncertain. All studies were at high overall risk of bias and provided conflicting evidence for the direction of association. Short-term NO_2_ exposure and the incidence of COVID-19 were considered to be positively associated, but uncertainly so. Most of the included studies showed associations in a positive direction, but all studies were at high overall risk of bias and most of them reported imprecise associations. A single study at low overall risk of bias [55] reported an imprecise positive association of short-term NO_2_ exposure with prevalence, providing a positive association of moderate certainty.

Unlike for mortality, a 10-μg/m^3^ increase (lag0–14) of O_3_ was associated with a 4.76% (95% CI: 1.99 to 7.52) increase in the daily counts of confirmed COVID-19 cases across 120 Chinese cities [59] and it correlated moderately with both daily new and total numbers of positive COVID-19 in Italy (Milan and Lombardy) [58]. A single study at high overall risk of bias indicated that short-term O_3_ exposure is positively associated with total cases, indicating moderate certainty of a positive association. Similarly, the association of short-term O_3_ exposure and incidence was reported by three studies, all at high overall risk of bias and all indicating positive associations. Two of these provided precise data, the remaining study indicated a potentially positive association. Given the study quality and varying precision of individual associations, this is also considered to be an uncertain positive association.

#### Effects of long-term air pollution on COVID-19 related-deaths

**Fig. 5:**
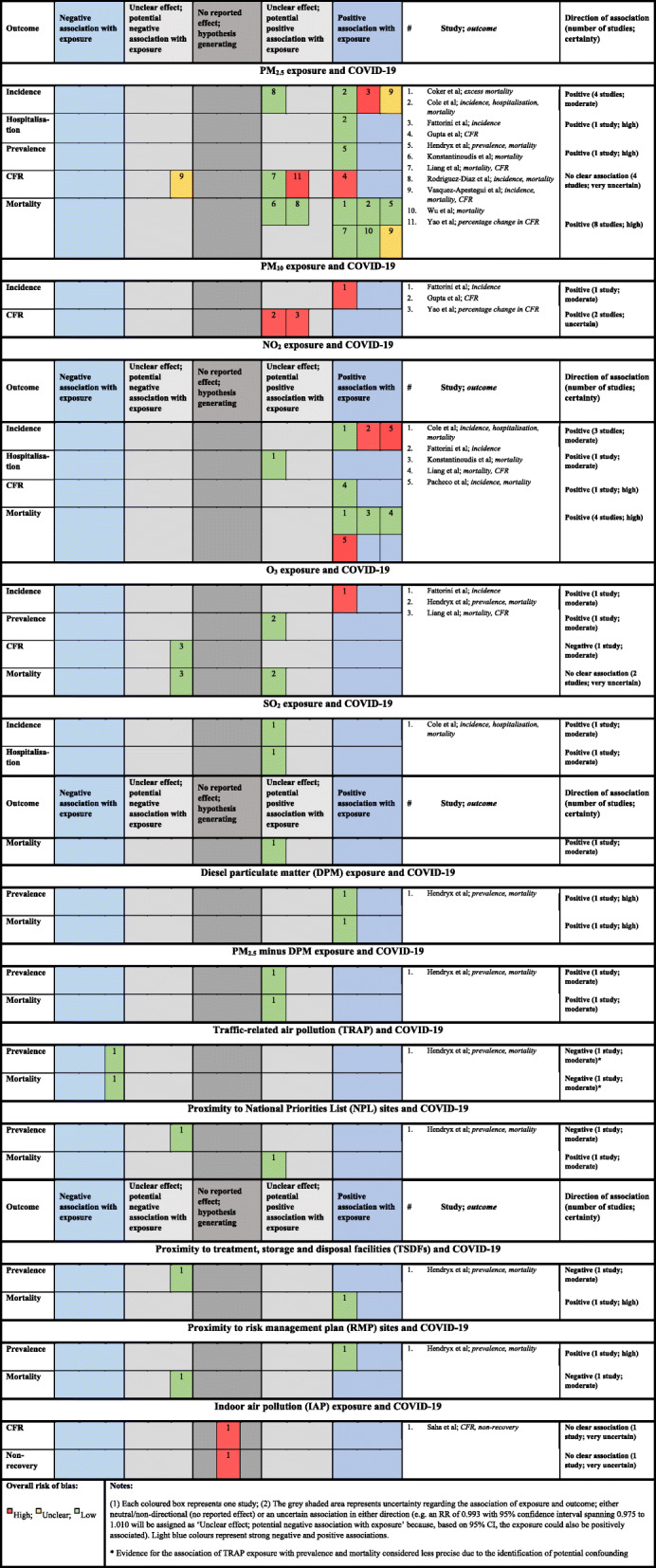
Harvest plots displaying level of evidence between long-term exposure to air pollution and risk, severity, incidence, and lethality for COVID-19 Pandemic

Table 2 displays the associations between long-term exposure to various pollutants and COVID-19 outcomes and Fig. 5 shows the related quality of evidence. Overall, an independent association has been described between long-term exposure to PM_2.5_ and COVID-19 related deaths. In Asia, estimates were reported from 49 Chinese cities where every 10 μg/m3 increase in PM_2.5_ concentrations was associated with 0.61% (0.09–1.12%) increase in CFR [52] and from nine Asian cities where long-term exposure to PM_2.5_ was reportedly associated with almost half of COVID-19 related-deaths [72]. In Europe, a nationwide study from England indicated every 1 μg/m^3^ increase in PM_2.5_ to be associated with COVID-19 mortality rate by 4.4% (3.7–5.1%), reduced to and 1.4% (− 2.1–5.1%) when adjusting for spatial autocorrelation and confounders [67]. Data from Northern Italy indicated every 1 μg/m3 increase in PM_2.5_ to be independently associated with 9% (95% CI: 6–12%) increase in excess mortality for COVID-19 [70]. In America, estimates from a cross-sectional nationwide in the USA using county-level data showed that every 1 μg/m^3^ increase in PM_2.5_ level was associated with an 11% (95% CI: 6, 17%) increase in COVID-19 death rate [69]. Similarly, a second cross-sectional nationwide in the USA using zero-inflated negative binomial models (adjusted for co-pollutants) showed that every 2.6 μg/m^3^ increase in PM_2.5_ concentration was marginally associated with 14.9% (95%CI: 0.0 to 31.9%) increase in mortality rate [68]. Besides, comparing predictors of COVID-19 cases and deaths between disproportionally Latino counties (> 17.8% Latino population) and all other counties in the USA, exposure to PM_2.5_ (third vs first quartile) was significantly associated with COVID-19 related-deaths (RR: 1.230; 95%CI 1.028, 1.471) [74]. Lastly, in Latin America, data from 24 districts in Lima (Peru) showed an independent association between PM_2.5_ and COVID-19 deaths per population density but not for CFR [56]. The association of long-term exposure to PM_2.5_ with CFR is very uncertain: two studies of better methodological quality [56, 68] indicated an unclear negative association; while two studies at high overall risk of bias [52, 72] indicated positive associations of varying precision. The association of long-term PM_2.5_ exposure with mortality shows high certainty of being positive. Overall, all studies contributing data were at low or unclear overall risk of bias, with two indicating imprecise positive associations and six showing precise positive associations.

**Table 2:** Association between long-term exposure to air pollution and risk, severity, incidence, and lethality for COVID-19 Pandemic

Study ID	Study Description	Outcomes	Main findings	Conclusion
Yao Y et al. [52], June 2020	*Associations between PM and CFR of COVID-19 *49 Chinese cities, spatial analysis	CFR	Pollutants (10 μg/m^3^ increase in and concentrations)- COVID-19 CFR increased by: *Long-term (2015–2019): • PM_2.5_: 0.61% (0.09–1.12%) and • PM_10_: 0.33% (0.03–0.64%) respectively.	PM pollution distribution and its association with COVID-19 CFR suggests that exposure to such may affect COVID-19 prognosis.
Hendryx M et al. [65], October 2020	Pollution data (PM_2.5_, DPM, O_3_) from the US Environmental Protection Agency Environmental Justice Screen, May 31, 2020 with 2014–2019	Cumulative prevalence and fatality rates	Estimate (SE), p-value. (Note: PM_2.5_ is one pollutant model. others, all indictors considered simultaneously) *Pollutants/ sources and COVID-19 Prevalence • PM_2.5_: 23.5, *p* = .02 • O_3_: 2.36 (3.29) *p* = .47 • Diesel PM: 237 (55.8) *p* = .001 • PM_2.5_minus DPM: 8.96 (10.8) *p* = .40 • Traffic: − 0.20 (.06) p = .02 • NPL sites: − 5.59 (113) *p* = .96 • TSDFs: − 1.75 (4.95) *p* = .72 • RMP sites: 56.7 (22.6) *p* = .01 *Pollutants/ sources and COVID-19 Death • PM_2.5_: 1.08 (.54) *p* = .05 • Ozone: 0.10 (.17) *p* = .54 • Diesel PM: 18.7 (2.80) p = .001 • PM_2.5_ minus DPM: 0.20 (.56) p = .72 • Traffic − 0.01 (.003) p = .001 • NPL sites: 3.76 (5.65) *p* = .51 • TSDFs: 0.52 (.25) *p* = .04 • RMP sites: − 0.83 (1.14) p = .47	Areas with worse prior air quality, especially higherconcentrations of diesel exhaust, may be at greater COVID-19 risk, although further studies are needed to confirm these relationships.
Fattorini D et al. [66], September 2020	Data on COVID-19 outbreak in Italian provinces and corresponding long-term air quality evaluations (four years), obtained from Italian and European agencies. Updated April 27, 2020	frequency and severity of cases (spread)	*Pollutants (average)-Incidence of COVID-19 • NO_2_: r = 0.4969, *p* < 0.01, (2016–2017) • PM_2.5_: r = 0.5827, p < 0.01, (2016–2017) • O_3_: r = 0.5142, p < 0.01 (2017–2016) • PM_10_: r = 0.4127, *p* < 0.05.(2017–2017) • PM_10_: r = 05168, p < 0.01 (2016–2017) *Long-term air-quality data significantly correlated with cases of COVID-19 in up to 71 Italian provinces	Atmospheric and environmental pollution should be considered as part of an integrated approach for sustainable development, human health protection and prevention of epidemic spreads but in a long-term
Konstantinoudis G et al. [67], December 2020	Long-term exposure to NO_2_ and PM_2.5_ (2014–2018 from the Pollution Climate Mapping) on COVID-19 deaths up to June 30, 2020 in England using high geographical resolution.	Death	Pollutants (1 μg/m3 increase)-COVID-19 Mortality rate: *Unadjusted • NO_2_: 2·6% (95%CrI: 2·4%-2·7%) • PM_2.5_: 4·4% (3·7%-5·1%) *Adjust for spatial autocorrelation and confounders • NO_2_: 0.5% (95% credible interval: − 0.2-1.2%) • PM_2.5_: 1.4% (− 2.1–5.1%).	some evidence of an effect of long-term NO_2_ exposure on COVID-19 mortality, while the effect of PM2.5 remains more uncertain
Liang D et al. [68], October 2020	Cross-sectional nationwide study using zero- inflated negative binomial models to estimate the association between long-term (2010–2016) county-level exposures to NO_2_, PM_2.5_ and O_3_ and county-level COVID-19 in the US.	CFR, Mortality	*Single Pollutant Model (estimate, 95%CI, *p*-value) COVID-19 CFR vs Mortality • NO_2_: 1.12, (1.05–1.18), 0.0003 vs 1.17, (1.10 to 1.25), < 0.0001 • PM_2.5_: 1.09, (0.96 to 1.23), 0.19 vs 1.19, (1.04 to 1.37), 0.012 • O_3_: 0.99, (0.93 to 1.06), 0.74 vs 1.00, (0.93 to 1.08), 0.95 *3- Pollutant Model (estimate, 95%CI, p-value) COVID-19 CFR vs Mortality • NO_2_: 1.11, (1.05 to 1.18), 0.0005 vs 1.16, (1.09 to 1.24), < 0.0001 • PM_2.5_: 1.06, (0.93 to 1.20), 0.39 vs 1.15, (1.00 to 1.32), 0.051 • O_3_: 0.98, (0.91 to 1.04), 0.48 vs 0.98, (0.91 to 1.05), 0.55 *Per IQR increase-COVID-19 CFR vs Mortality • NO_2_ (4.6 ppb): increase of 11.3% (95% CI 4.9 to 18.2%) vs 16.2% (95% CI 8.7 to 24.0%) • PM_2.5_ (2.6 μg/m^3^) marginally associated with 14.9% (95% CI 0.0 to 31.9%)increase mortality rate.	*Long-term exposure to NO_2_, which largely arises from urban combustion sources such as traffic, may enhance susceptibility to severe COVID-19 outcomes, independent of long-term PM_2.5_ and O_3_ exposure. *The results support targeted public health actions to protect residents from COVID-19 in heavily polluted regions with historically high NO_2_ levels.
Wu X et al. [69], November 2020	A nationwide, cross-sectional study using county-level data for long-term average exposure to PM_2.5_ and risk of COVID-19 death in the US (≥ 3000 counties, representing 98% of the population) up to April 22, 2020 from Johns Hopkins University	Mortality	PM_2.5_-COVID-19 Mortality: • MRR: 1.11 (1.06, 1.17) • 1 μg/m^3^ associated with an 11% (95% CI: 6, 17%) increase in death rate	*A small increase in long-term exposure to PM_2.5_leads to a large increase in the COVID-19 death rate. *Despite the ecological study design, importance of continuing to enforce existing air pollution regulations to protect human health both during and after the COVID-19 crisis.
Vasquez-Apestegui et al. [56], July 2020	Levels of PM_2.5_ exposure in the previous years (2010–2016) in 24 districts of Lima with the cases, deaths, and case-fatality rates of COVID-19.	Incidence, CFR and mortality	* PM_2.5_ (estimate, 95%CI) and COVID-19: • Case/population density: 0.070**, (0.034–0.107) • Death/ population density: 0.0014*, (0.0006–0.0023) • CFR: − 0.022, (− 0.067–0.023) Note: p < 0.05; **p < 0.01.	The higher rates of COVID-19 in Metropolitan Lima is attributable, among others, to the increased PM_2.5_ exposure in the previous years
Coker ES et al. [70], August 2020	Ecologic association between long-term concentrations of area-level of PM_2.5_ (2015–2019) and excess deaths in the first quarter of 2020 in municipalities of Northern Italy.	Excess mortality	* PM_2.5_ (estimate, SE)-COVID-19 Excess Deaths • No geographical effects: 0.128*** (0.008) • Regional fixed effects: 0.085*** (0.009) • LLS random effects: 0.089*** (0.014) • Regional fixed effects and LLS: 0.089*** (0.014) • 1 μg/m^3^ increase= > 9% (95% CI: 6–12%)*** increase in mortality. Note: ***p < 0.01, **p < 0.05, *p < 0.1	Positive association of ambient PM_2.5_ concentration on excess mortality in Northern Italy related to the COVID-19 epidemic.
Cole et al. [71], August 2020	Ecological association between long-term concentrations of of PM_2.5_ NO_2_, SO_2_ (2015–2019) and COVID-19 in 355 municipalities in Netherlands (National Institute for Public Health and the Environment)	Death, incidence and hospital admission	*Average 5 years (estimate, SE)=> COVID-19 cases: • PM_2.5_: 0.11*(0.051) • NO_2_: 0.027*(0.012) • SO_2_: 0.11 (0.079) COVID-19 admissions • PM_2.5_: 0.15*(0.065) • NO_2_: 0.015 (0.013) • SO_2_: 0.055 (0.065) COVID-19 deaths • PM_2.5_: 0.23**(0.073) • NO_2_: 0.035*(0.016) • SO_2_: 0.18 (0.10) Note: ***p < 0.001, **p < 0.01, *p < 0.05 Pollutants (1 μg/m3 increase)-COVID-19 Cases: • PM_2.5_: 9.4 (95%CI: 1.1,17.7) • NO_2_: 2.2 (95%CI: 0.2,4.3) Admissions • PM_2.5:_ 3.0 (95%CI: 0.43, 5.6) Deaths • PM_2.5:_ 2.3 (95%CI: 0.87,3.6) • NO_2_: 0.35 (95%CI: 0.042,0.66)	Relationship between COVID-19 and PM_2._5 persists even when a wide range of control variables are included and a number of different estimation methods used.
Gupta A et al. [72], July 2020	Data related to 9 Asian cities analysed to assess the link between mortality rate in the infected cases and the air pollution (WHO databases 2007–2016)	Mortality	Percentage of mortality per reported COVID-19 cases • Log10 (PM_2.5_): coef, SE, p: 5.747, 2.169, 0.033 • Log10 (PM_10_): coef, SE, p: 3.226, 1.811, 0.118 Percentage mortality per reported COVID-19 cases • PM_2.5_ (R^2^ = 50.1% and R^2^ Adj = 42.9%) • PM_10_ (R^2^ = 31.2% and R^2^ Adj = 24.1%).	Positive correlation indicating air pollution to be an elemental andconcealed factor in aggravating the global burden of deaths related to COVID-19
Pacheco H et al. [73], July 2020	Spatio-temporal variations in NO_2_ concentrations in 12 highly populated cities in Ecuador by comparing NO_2_ tropospheric concentrations before (March 2019) and after (March 2020) the COVID-19 lockdown.	Incidence, Mortality	NO_2_-COVID-19: • Cases: r = 0.88; p < 0.001 • Deaths: r = 0.91; *p* < 0.001 • Death per Capita: r = 0.84; *p* < 0.01	*Reduction in NO_2_ of up to 22–23% in the most highly populated cities in Ecuador (Quito and Guayaquil) after the lockdown caused by the outbreak of COVID-19. *Crucial role played by air quality as regards human health.
Saha J et al. [54], July 2020	Data from the 4th round of the National Family Health Survey 2015–16, and from the Ministry of Health and Family Welfare on 18th May 2020 to assess link between pre-existing morbidity conditions and IAP and COVID-19 among under-five children in India	Risk factor current fatality and recovery rate	Mean (SD) composite risk score of different indicators of indoor domestic smoky environment with COVID-19: • CFR: 2.5 (2.5) • Non-Recovery Rate: 47.5 (18.6)	From a research viewpoint, there is a prerequisite need for epidemiological studies to investigate the connection between indoor air pollution and pre-existing morbidity which are associated with COVID-19.
Rodriguez-Diaz CE et al. [74], July 2020	Comparison of predictors of COVID-19 cases and deaths between disproportionally Latino counties (> 17.8% Latino population) and all other counties through May 11, 2020.	Incidence, Death.	* PM_2.5_-COVID-19 Rate ratios (third vs. first quartile): • Cases: RR(95%CI): 1.028 (0.918, 1.151) • Deaths: RR(95%CI): 1.230 (1.028, 1.471)	Structural factors place Latino populations and particularly monolingual Spanish speakers at elevated risk for COVID-19 acquisition.

*Abbreviations*: *PM*_*2.5*_ *and*_*10*_ Particulate matter of diameter ≤ 2.5 and ≤ 10 μm respectively, *O*_*3*_ Ozone, *CO* Carbon monoxide, *SO*_*2*_ Sulfur dioxide, *NO*_*2*_ Nitrogen dioxide, *Pb* lead, *CH*_*4*_ Methane. *ICU* Intensive care unit, *CFR* Case fatality rate, *AQI* Air quality index, *VOC* Volatile organic compounds, *IQR* Interquartile range, *ACE-2* Angiotensin-Converting Enzyme 2, *IRR* Incidence rate ration. *US EPA* United States Environmental Protection Agency. *CI* Confidence Interval, *IAP* Indoor air pollution, *VS* Versus, *Log10* Logarithm to base 10, *RR* Rate ratio, *ppb* Part per billion (ppb), *r* coefficient of correlation, *Adj* Adjusted, *MRR* Mortality rate ratio. *DPM* Diesel particulate matter, *NPL* National Priority List, *TSDFS* Treatment, Storage or Disposal Facilities, *RMP* Risk Management Plan. *SD* Standard deviation, *SE* Standard error, *US* United States, *μg/m3* Microgram per cubic meter

For every 10 μg/m^3^ increase in PM_10_ concentrations, COVID-19 CFR increased by 0.33% (0.03–0.64%) [52] across 49 Chinese cities. Similarly, 24% of mortality among reported COVID-19 cases across nine cities in Asia were associated with exposure to PM_10_ but this was not statistically significant [72]. Two studies reporting associations of long-term exposure to PM_10_ and CFR were at high overall risk of bias and reported potentially positive associations, leading to an uncertain positive association. In the USA, a nationwide analysis showed that per every 8.95 μg/m^3^ increase in NO_2_ level, COVID-19 CFR and mortality increased by 11.3% (95% CI 4.9 to 18.2%) and 16.2% (95% CI 8.7 to 24.0%), respectively [68]. Similarly, an analysis considering spatio-temporal variation in NO_2_ concentrations in 12 highly populated cities in Ecuador also indicated a strong positive and significant correlation between NO_2_ and deaths (r = 0.91) [73]. In contrast, in England, every 1 μg/m^3^ increase in NO_2_ was associated with a nonsignificant change of only 0.5% (95% credible interval: − 0.2-1.2%) in COVID-19 mortality rate after adjusting for spatial autocorrelation and other confounders [67]. Long-term exposure to NO_2_ was positively associated with CFR in one study at low overall risk of bias [68], resulting in high certainty of a positive association of this exposure on CFR. Long-term exposure to NO_2_ was also positively associated with mortality, with a high degree of certainty. Three studies all indicate precise positive associations. Two of these studies were at low, and one study at high, overall risk of bias. Overall, studies failed to link chronic exposure to O3 with COVID-19 prevalence [65] or COVID-19 deaths in both studies conducted in the USA [65, 68] . However, a study across 71 Italian provinces did find a moderate positive and significant correlation between long-term exposure to O_3_ and COVID-19 incidence (r = 0.5142) [66]. The same study reported a potentially negative association of long-term O_3_ exposure on CFR, leading to moderate certainty of a potential negative association for this exposure and outcome. The overall association of long-term O_3_ exposure on mortality is very uncertain. Both studies contributing data were at low overall risk of bias and provided conflicting evidence for the direction of association.

#### Effects of long-term air pollution on COVID-19 susceptibility

A significant correlation between PM_2.5_ and COVID-19 incidence was observed in Italy [66], in Lima [56] and in the Netherlands [71] but not in the US when comparing Latino vs non-Latino counties (cases: RR 1.028; 95%CI 0.918, 1.151)) [74]. However, a significant correlations between PM_2.5_, diesel PM, traffic and COVID-19 prevalence were observed in the USA [65]. Long-term exposure to PM_2.5_ was shown by four studies to have positive associations, of varying precision, with incidence of infection. One study at low overall risk of bias [74] showed an unclear potentially positive effect, while three studies at high, unclear and low overall risk of bias [56, 66, 71] showed precise positive effects. This information provides moderately certain evidence of a positive association of PM_2.5_ exposure with incidence of COVID-19 over the long term. Exposure to PM_2.5_ showed a strong positive association with COVID-19 hospitalization from a single study at low overall risk of bias conducted in the Netherlands [71].

Across 71 Italian provinces, chronic exposure to PM_10_ significantly and moderately correlated with COVID-19 incidence [66]. A single study at high overall risk of bias indicated that long-term PM_10_ exposure is positively associated with incidence, indicating moderate certainty of a positive association. In addition, in Italy, Ecuador and the Netherlands, positive and moderate significant correlations were described between exposure to NO_2_ and incidence for COVID-19 [66, 71, 73]. Long-term exposure to NO_2_ was associated with an increase in the incidence of COVID-19, a finding of moderate certainty from two studies at high overall risk of bias and one study at low overall risk of bias. An uncertain positive association between NO_2_ exposure and hospitalization due to COVID-19 was observed from a single study at low overall risk of bias [71] . A single study at high overall risk of bias also indicated that long-term O_3_ exposure was positively associated with incidence, indicating moderate certainty of a positive association. The association of long-term O_3_ exposure and prevalence was positive and of moderate certainty, based on a single study at low overall risk of bias [65] suggesting an imprecise positive relationship. Finally, a single study at low overall risk of bias [71] indicated moderate certainty of positive associations between SO_2_ exposure and incidence, hospitalization and mortality.

## Discussion

This systematic review of the effects of short-and long-term exposure to air pollution in relation to the epidemiology and outcomes of COVID-19 included 25 primary studies. Overall, findings show that exposures to air pollutants, such as PM_2.5_, NO_2_ and to some extent PM_10_, O_3_, SO_2_, and CO, may have aggravated the health consequences of the COVID-19 pandemic. These findings provide a valuable additional reason in the plea for implementing or improving environmental policies, such as limiting emissions, to reduce the adverse effects of pollutants [62].

Firstly, in relation to short-term exposures, the association existing between particulate matter and COVID-19 CFR indicates that short-term increases, which are largely dependent on temporal changes in emissions and meteorology, can have an adverse effect on the prognosis of COVID-19 [52]. .The largest number of cases of COVID-19 was recorded in the most polluted regions, with patients presenting with more serious manifestations of disease needing ICU admissions. Mortality in these regions was twice as high as in the other regions. However, it was also found that although towns in Piedmont (Italy) had had the most severe PM10 emissions compared to Brescia and Bergamo in Lombardy (Italy), they had suffered fewer cases of infections [30]. Only a small correlation of NO_2_ levels was found in relation with SARS-CoV-2 prevalence, although at each time point a beneficial relation was apparent at higher amounts. Acute exposures to O_3_ may affect the transmission and initiation of COVID-19, but aggravation and mortality depend on other factors [60].

Secondly, considering long-term exposures, variations of which are essentially determined by spatial factors, findings showed a positive correlation of atmospheric PM_2.5_ concentration with COVID-19 excess mortality in Northern Italy [70]. Similarly, the incidence and severity of COVID-19 within Metropolitan Lima were associated, among other factors, to the degree of exposure of PM_2.5_ in previous years [56]. In the USA, one nationwide cross-sectional study concluded that a slight rise in chronic exposure to PM_2.5_ contributed to a significant increase in the mortality rate of COVID-19 [69] while another one reported only a marginal effect of PM_2.5_ in relation to COVID-19 susceptibility but not to mortality. The latter study, however, attributed much importance to the possible effects of long-term exposure to NO_2_ (primarily due to urban combustion sources such as traffic) on both mortality and susceptibility to severe COVID-19 regardless of long-term exposure to PM_2.5_ and O_3_ [68]. Similarly, a nationwide study in England, highlighted some evidence linking long-term exposure to NO_2_ to COVID-19 mortality while the effect of PM_2.5_ remained unclear [67]. Equally, a study from Lima reported a strong relationship between level of NO_2_ and confirmed cases/deaths caused by COVID-19 [73].

Thirdly, in China, PM_2.5_ and diurnal temperature closely correlated with COVID-19 deaths [64]. During photochemical pollution episodes, air pollutants (O_3_, PM_10_, and NO_2_) result from a combination of meteorological effects and chemical reactions. Air temperature influences the movement of air and thus the movement of air emissions. Considering that the atmosphere is the medium in which air pollutants are transported away from the source and that meteorological variables such as temperature and air pollutants vary daily, it is necessary to consider their relationship in the planetary boundary layer as one might directly influence another, and both are known to be associated with adverse health effects [75–78].

Correspondingly, across Latin American cities [63], humidity, wind speed and rainfall were also associated with COVID-19 outcomes. Further, in these cities, wage disparities (Gini index) and poverty levels were also linked to the spatial distribution of COVID-19. In the USA, ethnicity, areas with bad previous air quality, particularly higher levels of diesel exhaust, could be at higher risk for COVID-19 [65]. Based on a statistical analysis of the annual burning of fossil fuels in transport and the annual average concentrations of atmospheric, PM_2.5_, PM_10_, NO_2_ in the various states of India showed that homeless persons, vulnerable people, hawkers, roadside sellers, and others who are frequently subjected to vehicle emissions were at increased risk in the COVID-19 pandemic [61]. Caution should be exercised regarding O_3_ exposure in relation to COVID-19 pandemic, because most observations were done during the cold season (winter-early spring), when O_3_ levels are typically much lower than in summer [58].

Finally, the effect of lockdown regulation has shown a substantial reduction on air pollution [31]. This positive and indirect association between COVID-19 pandemic and air quality has echoed the need for cleaning air to protect human health both during and after the COVID-19 crisis [69, 79, 80].

These findings, however, need to be considered within the context of the methodological limitations of the studies that contributed data to this rapid review of the evidence. These methodological considerations are described comprehensively in the paper by Villeneuve & Goldberg [20], but certain key issues from our evidence base should be highlighted: 1/aggregated data from the majority of studies may not have included all groups to which findings could be generalized (such as institutionalized persons); 2/ addressing misclassification of outcomes, especially at the onset of the pandemic, likely biased associations with no clear direction (away from or towards the null); 3/accounting for the timing of measurements and where these occurred in relation to the pandemic curve or local lockdowns and other public health measures; 4/ although some studies considered lag effects as well as relative humidity and other pollutants, measuring differences in pollution levels across space and time should be an asset; 5/ considering the clustered nature of outcomes due to the infectious nature of the disease; 6/ adjusting for history of comorbidity and for COVID-19 genome mutations; and 7/ conducting Poisson or negative binomial models, which are more appropriate for count data rather than using models with normally distributed errors. We also acknowledge that ecological studies do not produce highly rigorous evidence and are prone to methodological fallacy and spurious associations [69]. We do, however, believe that systematically synthesizing the evidence, using our rapid adapted risk of bias tool and a conservative approach to assessing overall association and certainty, mitigates these concerns to a large extent, since better-conducted studies carried more weight in judging the overall association.

The evidence base was also limited in terms of its global generalizability, especially with regard to low-income countries. Thus our analysis did not include any study conducted in Africa, although evidence on adverse health effects for ambient air pollution is mounting in Africa [32]. The paucity of studies from low-income countries may also be important to assess the impact of household air pollution because biomass combustion contributes a lot to the population’s exposure to air pollution in poor countries [81]. Thus, only one study from India examined COVID-19 outcomes in relation to household air pollution and suggested the need of examining the association between COVID-19 and indoor air pollution with its associated pre-existing morbidity [54]. More studies are needed to address this question since the lockdowns, which led to improvements in outdoor air quality by reducing traffic and and industrial pollution [31, 33], may have had opposite effects for indoor air quality by leading to more burning of biomass fuel for domestic energy and more people smoking in the home [82].

In addition, we have included papers from the medRxiv [83–85] preprint, considered as non-peer-reviewed “grey literature” that were published in Medline but, where possible, we updated the information in January 2021 after the first round of peer-review [67–69].

Altogether, the limitations of our rapid systematic review might affect the current evidence in either direction. While our work outlines the need for updating data associating these two pandemics regularly to inform policy on their interplay in the global burden of diseases, it also highlights the quality of current available evidence. The high risk of bias observed in current studies calls for improving future studies [20, 86] and the constructive self-critique by Wu et al. [69] on the ecological design must be applauded as a useful contribution to the field. Consequently, the next generation of studies of the association between air pollution and COVID-19 epidemiology (particularly mortality related-outcomes) should prioritize obtaining individual-level data (or microdata) for both exposure (or using methods with a very high temporal and spatial resolution) and COVID-19 outcomes (or considering a control group of negative-tested patients).

## Conclusion

Although the evidence cannot be considered to be quite solid, our systematic review supports the view that air pollution has adversely influenced the COVID-19 related burden. Short-term and long-term exposures to PM_2.5_, and long-term exposures NO_2_ appear to be most consistently associated with COVID-19 epidemiological and clinical data worldwide, but studies assessing the effects of acute exposures presented substantial risks of bias. We call for future studies to focus on obtaining individual-level data for both exposure and COVID-19 outcomes. In the meantime, our findings encourage targeted global health actions integrating atmospheric and environmental pollution mitigation plans to sustain COVID-19 preparedness and responses.

## Supplementary Information

**Additional file 1: eTable 1**. Relationship between exposure to air pollution and COVID-19 Pandemic: an overview of published reviews. **eTable2.** Main characteristics of studies included in the systematic review for narrative summary. **eTable 3.** Risk of bias among studies assessing short-term exposure to air pollution in relation to COVID-19 outcomes. **eTable 4.** Risk of bias among studies assessing long-term exposure to air pollution in relation to COVID-19 outcomes. Harvest plot synthesis (results extension).

## Data Availability

The datasets supporting the conclusions of this article are included within the article (and its additional files).

## Abbreviations

WHO: World Health Organization
SARS-CoV-2: Severe acute respiratory syndrome coronavirus
TRAP: Traffic related air pollution
HAP: Household air pollution
ACE-2: Angiotensin-converting enzyme 2
ARI: Acute respiratory infection
COPD: Chronic obstructive pulmonary diseases
HPT: Hypertension
BMI: Body mass index
CFR: Case fatality rate
MR: Mortality rate
RoB: Risk of bias
ICD: International Classification of Dieases
GRADE: G*rading* of Recommendations, Assessment, Development and Evaluation
PM_2.5_ and _10_: Particulate matter of diameter ≤ 2.5 and ≤ 10 μm respectively
O_3_: Ozone
CO: Carbon monoxide
SO_2_: Sulfur dioxide
NO_2_: Nitrogen dioxide
Pb: Lead
CH_4_: Methane
ICU: Intensive care unit
AQI: Air quality index
VOCs: Volatile organic compounds
IQR: Interquartile range
IRR: Incidence rate ration
US EPA: United States Environmental Protection Agency
IAP: Indoor air pollution
DPM: Diesel particulate matter
NPL: National Priority List
TSDFS: Treatment, Storage or Disposal Facilities
RMP: Risk Management Plan
