# Supplementary material for: Acute and chronic exposure to air pollution in relation with incidence, prevalence, severity and mortality of COVID-19: a rapid systematic review
Source: Environ Health. 2021 Apr 10;20:41. doi: 10.1186/s12940-021-00714-1 (PMC8035877; doi:10.1186/s12940-021-00714-1)
Supplement: Supplementary file 1 — Additional file 1: eTable 1. Relationship between exposure to air pollution and COVID-19 Pandemic: an overview of published reviews. eTable2. Main characteristics of studies included in the systematic review for narrative summary. eTable 3. Risk of bias among studies assessing short-term exposure to air pollution in relation to COVID-19 outcomes. eTable 4. Risk of bias among studies assessing long-term exposure to air pollution in relation to COVID-19 outcomes. Harvest plot synthesis (results extension). [file 12940_2021_714_MOESM1_ESM.docx]

**SUPPLEMENTAL MATERIALS**

## eTable 1: Relationship between exposure to air pollution and COVID-19 Pandemic: an overview of published reviews

## eTable2. Main characteristics of studies included in the systematic review for narrative summary

## eTable 3: Risk of bias among studies assessing short-term exposure to air pollution in relation to COVID-19 outcomes

## eTable 4: Risk of bias among studies assessing long-term exposure to air pollution in relation to COVID-19 outcomes

## Harvest plot synthesis (results extension)

## **eTable 1: Relationship between exposure to air pollution and COVID-19 Pandemic: an overview of published reviews**

| **Studies** | **Main Findings** |
| --- | --- |
| Comunian et al., 2020(1) | Potential role of PM in the spread of COVID-19 in Italian cities which has higher PM level daily before the pandemic. |
| Moelling et al., 2020(2) | Air pollution linked to outcomes of SARS coronavirus (SARS-CoV) infections which may have contributed to severe SARS-CoV-2 outbreaks, especially in China, Northern Italy, Iran, and New York City. |
| Engin et al., 2020(3) | Smoking, air pollution and obesity are risk factors related to SARS-CoV-2 infection via the interference that NO_2_ increases ACE activity. |
| Bornstein et a., 2020(4) | Correlation between the level of environmental pollutants such as pesticides, dioxins, NO_2_ level which is effect metabolism and immune system with high rate of mortality by severe COVID-19 infection at European countries. |
| Tsatsakis et al., 2020(5) | Long-term exposure to chemicals in mixtures-mostly fossil fuel derivatives, particle matters, metals, ultraviolet (UV) and ionizing radiation contribute to immunotoxicity observed in COVID-19 pandemic. |
| Domingo et al., 2020a(6) | Clear correlation between some pollutants and human respiratory viruses, interaction to effect adversely the respiratory system. |
| Domingo and Rovira, 2020b(7) | Chronic exposure to air pollutants might lead to more severe and lethal forms of COVID-19 and complicates recovery of infected patients. |
| Al Huraimel et al., 2020(8) | Need for enough evidence at future for the association between air pollution and COVID-19 spread; air quality may be important parameter to evaluate the infectivity of COVID-19. |
| Chiara Copat et al., 2020(9) | Important contribution of PM_2.5_ and NO_2_ as triggering of the COVID-19 spread and lethality, and with a less extent also PM10, although the potential effect of airborne virus exposure it has not been still demonstrated. |

Abbreviations: PM_2.5_ and _10_: particulate matter of diameter ≤2.5 and ≤10 micrometers respectively, NO2: nitrogen dioxides; SARS-CoV-2: Severe acute respiratory syndrome coronavirus-2; ACE-2: Angiotensin-Converting Enzyme-2.

## **eTable2. Main characteristics of studies included in the systematic review for narrative summary**

| **Author** | **Publication year** | **Region** | **Study design** | **Total cases/Total deaths** | **Study Period** | **Exposure assessment method** | **Pollutant** | **Adjustment variables** |
| --- | --- | --- | --- | --- | --- | --- | --- | --- |
| A.Adhikari(10) | 5 June 2020 | New York, USA | Retrospective cohort | 42.023 cases, 3221deaths | March – April 2020 | Regression modeling | O_3_, PM_2.5_ |  |
| M.F. Bashir(11) | 13 May 2020 | California, USA | Cross-sectional | 39,000 cases, including >1800 deaths. | March – April 2020 | Correlations (Spearman, Kendall) | PM_2.5_, PM_10_, SO_2_, NO_2_, Pb, VOC |  |
| Bolano(12) | 10 July 2020 | Latin America, Caribbean region | Retrospective cohort |  |  |  | PM_10_, PM_2.5_, NO_2_ |  |
| Bontempi(13) | 07 May 2020 | Lombardi and Piedmont, Italy | Retrospective cohort |  | February - March, 2020 | Correlations | PM_10_ |  |
| Marina Boro(14) | 2 August 2020 | 110 Provinces of Italy | Retrospective cohort |  | February – March 2020 | Correlation analysis | PM_2.5_ |  |
| Chakraborty(15) | 15 July 2020 | Cities in India | Cross-sectional |  |  | Correlation analysis | PM_2.5_, PM 10, NO_2_ |  |
| Eric S. Coker(16) | 4 August 2020 | municipalities of Northern  Italy | Ecological |  | First quarter of March | The negative binomial models | PM_2.5_ |  |
| Edoardo Conticini(17) | 4 April 2020 | Northern Italy | Cross-sectional |  |  | Correlation analysis | PM_10_, PM_2.5_, O_3_, SO_2_ and NO_2_ |  |
| Daniele Fattorini(18) | 4 May 2020 | Northern Italy | Retrospective cohort | 20.000 cases | February – April 2020 | Correlation analysis | NO_2_, O_3_, PM_2.5_ and PM_10_ |  |
| Tommaso Filippini(19) | 16 June 2020 | Lombardy, Veneto and Emilia-Romagna regions, Italy | Ecological (Time series) |  | Mars – April 2020 | Multivariable restricted cubic Spline regression model | NO_2_ |  |
| Antonio Frontera(20) | 21 May 2020 | Italy | Cross-sectional |  |  | Pearson correlation analysis | PM_2.5_ and NO_2_ |  |
| Ankit Gupta(21) | 9 July 2020 | Cities from India, China, Pakistan | Cross-sectional |  | March – July 2020 | Linear regression model | PM_2.5_, PM_10_ |  |
| Michael Hendryx(22) | 27 June 2020 | USA | Retrospective cohort |  | March – May | Pearson correlation | PM_2.5_, O_3_, Diesel PM, Traffic. | percent of the population over age 65; percent  race/ethnicity groups (African American, Asian, Native American/  Pacific Islander, Hispanic, and non-Hispanic White (used as the  referent in regression models)); percent of adults with at least  some college education; income inequality (the ratio of household  income at the 80th percentile to that at the 20th percentile); adult  smoking rate; adult obesity rate; and percent of the population  without health insurance. |
| Ying Jiang(23) | 5 August 2020 | Wuhan, China | Retrospective cohort |  | January – April 2020 | Poisson regression model | PM_2.5_, PM_10_, SO_2_, CO, NO_2_, and O_3_ |  |
| Garyfallos Konstantinoudis(24,25) | August 11, 2020 | England, UK | Ecological | 38 573 deaths | March – June 2020 | Bayesian hierarchical models to quantify the effect of air-pollution | NO_2_ and PM_2.5_ | Temperature, Relative humidity Index of Multiple Deprivation, Urbanicity, days since 1^st^ reported case, number of positive cases, population density, Number of intensive care unit beds, smoking, Obesity, High risk occupation |
| He Li(26) | 20 May 2020 | Wuhan and XiaoGan, China | Retrospective cohort |  |  | Correlation analysis | PM_2.5_, PM_10_, NO_2_ and CO) | daily temperature, highest temperature, lowest temperature, temperature difference and sunshine duration |
| Donghai Liang(27,28) | May 7, 2020 | USA | Cross-sectional | 1,027,799 cases / 58,489 deaths |  | Zero-inflated negative binomial mixed models | NO_2_, PM_2.5_ and O_3_ | county-level healthcare capacity, population mobility, sociodemographic,  SES, behavior risk factors, and meteorological factors. |
| Henry Pacheco(29) | 20 July 202 | Ecuador | Cross-sectional |  |  | Pearson's correlation | NO_2_, SO_2_, CO, CH4 and O_3_ |  |
| Loredana Raciti(30) | 26 June 2020 | Messina, Italy | Hypothesis |  |  |  |  |  |
| Jay Saha(31) | 15 July 2020 | India | Cross-sectional |  |  |  |  |  |
| Matthew Cole(32) | 4 August,2020 | The Netherlands | Ecological | Mean(minimum-maximum) cases: 131.46(0-2416), hospital admissions: 33.02(0-611), deaths: 16.87(0-336) | February-June 2020 | Negative  binomial model | PM_2.5,_ NO_2_ and SO_2_ | A number of variables including instrumenting pollution to mitigate potential  endogeneity in the measurement of pollution and modelling spatial spillovers using spatial econometric techniques. |
| Xiao Wu(33,34) | April 27,2020 | USA | Cross-sectional | 45,817 deaths | March – April 2020 | Negative binomial mixed model | PM_2.5_ | population size, age distribution, population density, time since the beginning of the outbreak, time since state’s issuance of stay-at-home order, hospital beds, number of individuals tested, weather, and socioeconomic and behavioral variables such as obesity and smoking |
| Yaqi Wang(35) | 6 June 2020 | Beijing, China | Ecological |  |  | Linear regression  models with penalized splines on NO2 | NO_2_ |  |
| Ye Yao(36) | 20 June 2020 | in 49 Chinese cities, including the epicenter of Wuhan | Cross-sectional |  | From January 15, 2020  to February 29, 2020 to | Multivariate linear regression | PM_10_, PM_2.5_ |  |
| Yongjian Zhu(37) | 15 April 2020 | In 120 cities in China | Retrospective cohort | 79,968 cases | From January 23, 2020 to February 29, 2020 | Generalized additive  Model | PM_2.5_, PM_10_, SO_2_, CO, NO_2_ and O_3_ |  |
| Maria A. Zoran(38) | 5 June 2020 | Milan metropolitan area and Lombardy region, Italy | Ecological (Time series) |  |  | Correlation analysis | O_3_ and NO_2_ |  |

Abbreviations: PM_2.5_ and _10_: particulate matter of diameter ≤2.5 and ≤10 micrometers respectively; O3: ozone; CO: carbon monoxide; SO2: sulfur dioxide; NO2: Nitrogen dioxide; Pb: lead; CH4: methane

## **eTable 3: Risk of bias among studies assessing short-term exposure to air pollution in relation to COVID-19 outcomes**

| **Study ID** | **Objective assessment of outcome** | **Adjustment for confounding** | **Use of control/dose-response comparator** | **Overall RoB judgment** |
| --- | --- | --- | --- | --- |
| Yao Y et al(36), June 2020 | Low risk: data on COVID-19 deaths were obtained from the National Health Commission and the Provincial Health Commissions of China; CFR is an objective outcome | High risk: study reports aggregated level of exposure and adjusts only for GDP and hospital beds per capita | Low risk: change in CFR for every 10 μg/m^3^ increase in pollutants (multivariate linear regression) | High |
| Frontera A et al(20), August 2020 | Low risk: data are official government data from the Italian Civil Protection website; hospitalization, ICU admission and death are objective outcomes | Unclear risk: study reports aggregated level of exposure and only weights regions according to the number of people > 65 years | Low risk: correlation of outcomes with pollutant levels in various regions (Pearson’s correlation) | Unclear |
| Li H(26) et al, August 2020 | Low risk: laboratory and clinical diagnoses of COVID-19 from CDC of Hubei Province | High risk: study reports aggregated level of exposure and does not adjust for age, sex, or any other covariate | Low risk: correlation of outcomes with pollutant levels (linear regression) | High |
| Zoran MA(38), October 2020 | Low risk: aggregated regional data from the Italian Health Ministry; total deaths is an objective measure | High risk: study reports aggregated level of exposure and does not adjust for age, sex, or any other covariate | Low risk: correlation of outcomes with pollutant levels (Pearson’s correlation reported, Spearman’s and Kendall’s correlations also explored) | High |
| Zhu Y et al(37), Jully 2020 | Low risk: reports released by local health commissions | High risk: study reports aggregated level of exposure and does not adjust for age or sex. Adjustment was done for other pollutants and lag effect | Low risk: change in confirmed daily cases for every unit increase in pollutants (10 μg/m^3^ for PM_2.5_, PM_10_, SO_2_, NO_2_ and O3; 1 mg/m^3^ for CO) using a generalized additive model | High |
| Adhikari A et al(10), Jun 2020 | Low risk: numbers of COVID-19 cases and related deaths obtained from a non-for-profit, nonpartisan civic initiative listing government agencies as its data source; mortality is an objective measure | High risk: study reports aggregated level of exposure and does not adjust for age or sex. Adjustment was done for lagged outcome and day trend | Low risk: single-predictor regression model correlating unit increases in pollutants with exponential form of IRR | High |
| Chakraborty P et al(15), July 2020 | Low risk: numbers of COVID-19 cases and related deaths obtained from state bulletins and official handles; mortality is an objective measure | High risk: study reports aggregated level of exposure and does not adjust for age, sex, or any other covariate | Low risk: correlation of outcome with pollutant levels/ratios (Pearson’s correlation) | High |
| Bontempi E(13), July 2020 | Low risk: numbers of COVID-19 cases obtained from the website of the Italian Department of Civil Protection – Presidency of the Council of Ministers | High risk: study reports aggregated level of exposure and does not adjust for age, sex, or any other covariate | High risk: no formal comparative analysis; narrative parallel comparison of [PM_10_] and COVID-19 incidence | High |
| Bashir MF et al(11), May 2020 | Low risk: numbers of COVID-19 cases and mortality obtained from California Department of Public Health; mortality is an objective measure | High risk: study reports aggregated level of exposure and does not adjust for age, sex, or any other covariate | Low risk: correlation of outcome with pollutant levels (Spearman’s and Kendall’s correlations) | High |
| Bolaño-Ortiz TR et al(12), July 2020 | Unclear risk: it is not clear where number of COVID-19 cases and related mortality was obtained from | High risk: study reports aggregated level of exposure and does not adjust for age, sex, or any other covariate | Low risk: correlation of outcome with pollutant levels (Spearman’s correlation) | High |
| Borro M et al(39), August 2020 | Low risk: numbers of COVID-19 cases obtained from the Italian Civil Protection Department database; mortality is an objective measure | High risk: study reports aggregated level of exposure and does not adjust for age or sex. Adjustments were done for outcomes normalized by incidence, mortality, and case fatality risk, as well as lag effect (comparing outcomes to pollutant levels four/five days earlier) | Low risk: change in confirmed daily cases for every unit increase in pollutants (linear regression) | High |
| Raciti L et al(30), Jun 2020 | Unclear: no information on where COVID-19 data were obtained from | High risk: study reports aggregated level of exposure and does not adjust for age, sex, or any other covariate | High risk: no formal comparative analysis; narrative hypothesis-generating discussion | High |
| Jiang Y et al(23), 2020 August | Low risk: COVID-19 mortality numbers obtained from the Health Commission of Hubei China (Hubei Province); mortality is an objective measure | High risk: study reports aggregated level of exposure and does not adjust for age or sex. Adjustment was done for an 18-day lag effect | Low risk: correlation of outcome with pollutant levels (Pearson and Poisson regression) | High |
| Filippini T et al(19), October 2020 | Low risk: regional and provincial data from the Italian national government | Low risk: study adjusts for population-level age ≥ 65 years, in addition to population density, mobility, the presence of an airport, as well as lagged temperature and relative humidity but reports aggregated level of exposure | Low risk: examination of relationship between outcome and pollutant levels using multivariable model restricted cubic spline regression | Low |

Abbreviations: PM_2.5_ and _10_: particulate matter of diameter ≤2.5 and ≤10 micrometers respectively; O_3_: ozone; CO: carbon monoxide; SO_2_: sulfur dioxide; NO_2_: Nitrogen dioxide; Pb: lead; CDC: centers for disease control and prevention; IRR: incidence rate ratio.

## **eTable 4: Risk of bias among studies assessing long-term exposure to air pollution in relation to COVID-19 outcomes**

| **Study ID** | **Objective assessment of outcome** | **Adjustment for confounding** | **Use of control/dose-response comparator** | **Overall RoB judgment** |
| --- | --- | --- | --- | --- |
| Yao Y et al(36), June 2020 | Low risk: data on COVID-19 deaths were obtained from the National Health Commission and the Provincial Health Commissions of China; CFR is an objective outcome | High risk: study reports aggregated level of exposure and adjusts only for GDP and hospital beds per capita | Low risk: change in CFR for every 10 μg/m^3^ increase in pollutants | High |
| Hendryx M et al(22), October 2020 | Low risk: COVID-19 prevalence and fatality rates were obtained from the Johns Hopkins University-run data repository including information from US state and county/city administrative departments; CFR is an objective outcome | Low risk: study adjusts for population-level age ≥ 65 years, ethnicity, education, income inequality, smoking, adult obesity, and percentage of population without health insurance; according to County Health Rankings but reports aggregated level of exposure | Low risk: association of outcome with pollutant levels (linear multiple regression) | Low |
| Fattorini D et al(18), September 2020 | Low risk: COVID-19 cases and deaths obtained from official daily reports of the Italian Civil Protection Department | High risk: study reports aggregated level of exposure and does not adjust for age, sex, or any other covariate | Low risk: correlation of outcome with pollutant levels (Pearson’s correlation) | High |
| Konstantinoudis G et al(24), August 2020 medRxiv and December 2020(25) | Low risk: COVID-19 deaths were obtained from Public Health England; mortality is an objective measure | Low risk: study reports aggregated level of exposure; but the authors report that all models were adjusted for age, sex and ethnicity using indirect standardization. Deprivation, urbanicity, population density and occupational exposure were considered as additional confounders. Temperature and relative humidity were population-weighted | Low risk: association of outcome with pollutant levels (Bayesian hierarchical Poisson log-linear models) | Low |
| Liang D et al(27), May 2020 medRxiv and October 2020(28) | Low risk: COVID-19 cases and mortality obtained from three sources; New York Times, USAFACTS and coronavirus.1point3acres.com/en (all aggregate data from official government websites and agencies); mortality is an objective measure | Low risk: study reports aggregated level of exposure; but the authors report that models were adjusted for age (percentage ≥ 60 years), male gender, population density, BMI, smoking rate, ICU and hospital beds, active medical doctors per 1000 people, travel mobility index, various socioeconomic indicators, air temperature and relative humidity | Low risk: association of outcome with pollutant levels (zero-inflated negative binomial mixed models (ZINB)) | Low |
| Wu X et al(33), April 2020, medRxiv and November 2020(34) | Low risk: COVID-19 mortality obtained from Johns Hopkins  University, Center for Systems Science and Engineering Coronavirus Resource Center (collating data from the CDC and state health departments) | Low risk: study reports aggregated level of exposure; but the authors report that models were adjusted for age (percentage ≥ 65 years, 45-64 years and 15-44 years), days since first COVID-19 case reported, population density, poverty, income, ethnicity, housing and house value, adult education, obesity, current smoking, number of hospital beds per unit population, average daily temperature, relative humidity and days since lockdown | Low risk: association of outcome with pollutant levels (binomial mixed models) | Low |
| Vasquez-Apestegui et al(40), July 2020 | Unclear: no information on where COVID-19 data were obtained from | Low risk: study reports aggregated level of exposure; but the authors report that models were adjusted for sex (female: male cases), age at COVID-19 diagnosis, PM_2.5_ level, food markets per district | Low risk: association of outcome with pollutant levels (linear regression) | Unclear |
| Coker ES et al(16), August 2020 | Low risk: excess mortality from COVID-19 obtained from the Italian National Statistical Institute and COVID-19 deaths obtained from the Italian Civil Protection Department; mortality is an objective measure | Low risk: study reports aggregated level of exposure; but the authors report that models were adjusted for gender, percentage aged > 65 years, excess deaths, PM_2.5_, population density, per capita income, industrial land, shared enterprises, temperature, non-EU residents, university students, distance to airport, total population and hospital beds per capita | Low risk: association of outcome with pollutant levels (negative binomial regression) | Low |
| Gupta A et al(21), July 2020 | Low risk: no information on where COVID-19 data were obtained from, but mortality is an objective measure | High risk: study reports aggregated level of exposure and does not adjust for age, sex, or any other covariate | Low risk: association of outcome with pollutant levels (linear regression) | High |
| Pacheco H et al(29), July 2020 | Low risk: COVID-19 cases and deaths were obtained from the Ecuadorian  Ministry of Public Health's public records | High risk: study reports aggregated level of exposure and does not adjust for age, sex, or any other covariate | Low risk: association of outcome with pollutant levels (simple regression and Pearson’s correlation) | High |
| Saha J et al(31), July 2020 | Low risk: COVID-19 fatality and recovery rates were obtained from the Ministry of Health and Family Welfare of the Government of India | Low risk: study reports individual-level exposure determined by survey, but does not adjust for age, sex, or any other covariate | High risk: no comparative analysis, using environmental factors with CFR and failed recovery rates to calculate mean composite risk Z scores | High |
| Rodriguez-Diaz CE et al(41), July 2020 | Low risk: COVID-19 cases and mortality obtained from USAFACTS (aggregated data from official government agencies); mortality is an objective measure | Low risk: study reports aggregated level of exposure; but the authors report that models were adjusted for age (percentage < 35 years), percentage Latinx, other ethnicity, employment, insurance, occupancy, lingualism, diabetes, heart disease fatality, cerebrovascular/hypertension fatality, HIV infection rate, urbanicity, PM_2.5_, social distancing and days since first case | Low risk: association of outcome with pollutant levels (multivariable analysis) | Low |
| Cole MA et al(32), August 2020 | Low risk: cross-sectional data provided by the National Institute for Public Health and the Environment (RIVM) | Low risk: study adjusts for residential address/geographical location, household income, household size, age, smoking, ethnicity, size of municipality and other explanatory variables, but reports aggregated level of exposure | Low risk: correlation of outcomes with pollutant levels (negative binomial model) | Low |

Abbreviations: PM_2.5_: particulate matter of diameter ≤2.5 micrometers CDC: centers for disease control and prevention; CFR: case fatality rate, BMI: body mass index; EU: European Union.

## **5.Harvest plot synthesis (results extension)**

Short-term exposure to SO_2_ showed an independent negative association with COVID-19 deaths in Wuhan (IRR (95%CI): 0.951: 0.919–0.984)(23) and in California (r= -0.397)(11). Two studies at high overall risk of bias indicated that short-term SO_2_ exposure is negatively associated with mortality, indicating moderate certainty of a negative association. While short term exposure to VOCs did not show effects on COVID-19 mortality in California, a positive but weak correlation was observed between exposure to lead and COVID-19 mortality in California (r=0.174)(11). The association of short-term VOC exposure and mortality is uncertain, with a single study at high overall risk of bias providing an imprecise positive association. A single study at high overall risk of bias indicated that short-term lead exposure is positively associated with mortality, indicating moderate certainty of a positive association. The overall association of short-term CO exposure with mortality is very uncertain. Both studies that contributed data were at high overall risk of bias and provided conflicting evidence for the direction of association.

For every 10-μg/m^3^ increase (lag0-14) of SO_2_, there was a decrease in COVID-19 confirmed cases of 7.79%: (95% CI: -14.57 to -1.01) in China(42). A negative correlation was also observed in California (r= - 0.426)(11). The overall association of short-term SO_2_ with incidence is very uncertain. Both studies contributing data were at high overall risk of bias and provided conflicting evidence for the direction of association. A protective effect was observed between short-term exposure to CO and COVID-19 mortality in Wuhan (RR, 95%CI: 0.177, 0.131–0.24)(23) but not in California(11). All studies reporting associations of short-term exposure to CO and incidence were at high overall risk of bias and reported positive associations of varying precision, leading to an uncertain positive association. While short-term exposure to VOCs did not show effects on COVID-19 incidence in California, a positive but weak correlation was observed between exposure to lead and COVID-19 incidence in California (r=0.178)(11). Volcanic gases and heavy metals-related air pollution, combined to specific climatic conditions and regional topography, favored severe COVID-19 diffusion in Sicily (Italy)(30). The association of short-term VOC exposure and incidence is uncertain, with a single study at high overall risk of bias providing an imprecise positive association. A single study at high overall risk of bias indicated that short-term lead exposure is positively associated with incidence, indicating moderate certainty of a positive association. Short-term exposure to higher AQI (corresponding with lower air quality) was associated with an increase in the incidence of COVID-19, a finding of moderate certainty from two studies reported in a single paper(26) at high overall risk of bias. Volcanic gases and heavy metal air pollution was hypothesized to be associated with COVID-19 incidence(30), but no formal analysis was conducted. Consequently, no association is reported.

Study reported an imprecise positive association of long-term exposure to PM_2.5_ without DMP and mortality, leading to moderate certainty of a potential positive association. This association did not hold for long-term exposure to traffic-related air pollution (TRAP) and mortality, however, since Hendryx and colleagues reported a precise negative association – the authors do report that this is likely due to inclusion of DPM and population density, both of which were moderately positively associated with TRAP, in the model. Therefore, the negative association is considered to be of moderate certainty due to the potential confounding.

The association of mortality with long-term proximity to National Priorities List (NPL) sites and treatment, storage and disposal facilities (TSDFs) was found to be positive, with moderate and high certainty, respectively. In contrast, the association of mortality with long-term proximity to risk management plan (RMP) sites was negative, with moderate certainty. All these findings were from a single study at low overall risk of bias(22).

While we did not find a study reporting the effect of chronic exposure to SO2 with regard to any COVID-19 outcomes, chronic exposure to pollutants such as diesel, traffic were associated with COVID-19 mortality in the USA(22). Long-term exposure to diesel particulate matter was positively associated with mortality in one study at low overall risk of bias(22), resulting in high certainty of a positive association of this exposure on mortality. One study at low overall risk of bias(22) reported precise positive association of long-term PM_2.5_ exposure and prevalence, providing a positive association of high certainty. Long-term exposure to diesel particulate matter was positively associated with prevalence in one study at low overall risk of bias(22), resulting in high certainty of a positive association. The same study reported an imprecise positive association of long-term exposure to PM_2.5_ minus DPM with prevalence, indicating a potential positive association of moderate certainty. Long-term exposure to traffic-related air pollution (TRAP) and mortality, however, were negatively associated according to this study.

**References**

1. Comunian S, Dongo D, Milani C. Air Pollution and COVID-19 : The Role of Particulate Matter in the Spread and Increase of COVID-19 ’ s Morbidity and Mortality. 2.

2. Moelling K, Broecker F. Air Microbiome and Pollution : Composition and Potential Effects on Human Health , Including SARS Coronavirus Infection. 2020;2020(X).

3. Basak A, Doruk E, Engin A. Two important controversial risk factors in SARS-CoV-2 infection : Obesity and smoking. Environmental Toxicology and Pharmacology. 2020;78(May):103411.

4. Bornstein SR, Voit-bak K, Schmidt D, Morawietz H, Bornstein AB, Balanzew W, et al. Is There a Role for Environmental and Metabolic Factors Predisposing to Severe COVID-19 ? Authors Is There any Other Plausible Explanation ? 2020;

5. Tsatsakis A, Petrakis D, Konstantinos T, Oana A, Calina D, Vinceti M, et al. COVID-19 , an opportunity to reevaluate the correlation between long-term effects of anthropogenic pollutants on viral epidemic / pandemic events and prevalence. Food and Chemical Toxicology. 2020;141(April):111418.

6. Domingo JL, Marquès M, Rovira J. Influence of airborne transmission of SARS-CoV-2 on COVID-19 pandemic. A review. Vol. 188, Environmental Research. Academic Press Inc.; 2020. p. 109861.

7. Domingo JL, Rovira J. Since January 2020 Elsevier has created a COVID-19 resource centre with free information in English and Mandarin on the novel coronavirus COVID- 19 . The COVID-19 resource centre is hosted on Elsevier Connect , the company ’ s public news and information . 2020;(January).

8. Huraimel K Al, Alhosani M, Kunhabdulla S, Stietiya MH. Since January 2020 Elsevier has created a COVID-19 resource centre with free information in English and Mandarin on the novel coronavirus COVID- 19 . The COVID-19 resource centre is hosted on Elsevier Connect , the company ’ s public news and information . 2020;(January).

9. Copat C, Cristaldi A, Fiore M, Grasso A, Zuccarello P, Signorelli SS, et al. The role of air pollution (PM and NO2) in COVID-19 spread and lethality: A systematic review. Environ Res. 2020 Dec;191:110129.

10. Adhikari A, Yin J. Short-Term Effects of Ambient Ozone, PM2.5, and Meteorological Factors on COVID-19 Confirmed Cases and Deaths in Queens, New York. Int J Environ Res Public Health [Internet]. 2020 Jun [cited 2020 Aug 2];17(11). Available from: https://www.ncbi.nlm.nih.gov/pmc/articles/PMC7312351/

11. Bashir MF, Ma BJ, Bilal null, Komal B, Bashir MA, Farooq TH, et al. Correlation between environmental pollution indicators and COVID-19 pandemic: A brief study in Californian context. Environ Res. 2020;187:109652.

12. Bolaño-Ortiz TR, Camargo-Caicedo Y, Puliafito SE, Ruggeri MF, Bolaño-Diaz S, Pascual-Flores R, et al. Spread of SARS-CoV-2 through Latin America and the Caribbean region: A look from its economic conditions, climate and air pollution indicators. Environ Res. 2020 Jul 15;191:109938.

13. Bontempi E. First data analysis about possible COVID-19 virus airborne diffusion due to air particulate matter (PM): The case of Lombardy (Italy). Environ Res. 2020 Jul;186:109639.

14. Boros PW, Lubiński W. Health state and the quality of life in patients with chronic obstructive pulmonary disease in Poland: a study using the EuroQoL-5D questionnaire. Pol Arch Med Wewn. 2012;122(3):73–81.

15. Chakraborty P, Jayachandran S, Padalkar P, Sitlhou L, Chakraborty S, Kar R, et al. Exposure to Nitrogen Dioxide (NO2) from Vehicular Emission Could Increase the COVID-19 Pandemic Fatality in India: A Perspective. Bull Environ Contam Toxicol. 2020 Aug;105(2):198–204.

16. Coker ES, Cavalli L, Fabrizi E, Guastella G, Lippo E, Parisi ML, et al. The Effects of Air Pollution on COVID-19 Related Mortality in Northern Italy. Environ Resour Econ (Dordr). 2020 Aug 4;1–24.

17. Conticini E, Frediani B, Caro D. Can atmospheric pollution be considered a co-factor in extremely high level of SARS-CoV-2 lethality in Northern Italy? Environ Pollut. 2020 Jun;261:114465.

18. Fattorini D, Regoli F. Role of the chronic air pollution levels in the Covid-19 outbreak risk in Italy. Environ Pollut. 2020 Sep;264:114732.

19. Filippini T, Rothman KJ, Goffi A, Ferrari F, Maffeis G, Orsini N, et al. Satellite-detected tropospheric nitrogen dioxide and spread of SARS-CoV-2 infection in Northern Italy. Sci Total Environ. 2020 Oct 15;739:140278.

20. Frontera A, Cianfanelli L, Vlachos K, Landoni G, Cremona G. Severe air pollution links to higher mortality in COVID-19 patients: The “double-hit” hypothesis. J Infect. 2020 Aug;81(2):255–9.

21. Gupta A, Bherwani H, Gautam S, Anjum S, Musugu K, Kumar N, et al. Air pollution aggravating COVID-19 lethality? Exploration in Asian cities using statistical models. Environ Dev Sustain. 2020 Jul 15;1–10.

22. Hendryx M, Luo J. COVID-19 prevalence and fatality rates in association with air pollution emission concentrations and emission sources. Environ Pollut. 2020 Oct;265(Pt A):115126.

23. Jiang Y, Xu J. The association between COVID-19 deaths and short-term ambient air pollution/meteorological condition exposure: a retrospective study from Wuhan, China. Air Qual Atmos Health. 2020 Aug 15;1–5.

24. Konstantinoudis G, Padellini T, Bennett JE, Davies B, Ezzati M, Blangiardo M. Long-term exposure to air-pollution and COVID-19 mortality in England: a hierarchical spatial analysis. medRxiv. 2020 Aug 11;

25. Konstantinoudis G, Padellini T, Bennett J, Davies B, Ezzati M, Blangiardo M. Long-term exposure to air-pollution and COVID-19 mortality in England: A hierarchical spatial analysis. Environ Int. 2021 Jan;146:106316.

26. Li H, Xu X-L, Dai D-W, Huang Z-Y, Ma Z, Guan Y-J. Air pollution and temperature are associated with increased COVID-19 incidence: A time series study. Int J Infect Dis. 2020 Aug;97:278–82.

27. Liang D, Shi L, Zhao J, Liu P, Schwartz J, Gao S, et al. Urban Air Pollution May Enhance COVID-19 Case-Fatality and Mortality Rates in the United States. medRxiv. 2020 May 7;

28. Liang D, Shi L, Zhao J, Liu P, Sarnat JA, Gao S, et al. Urban Air Pollution May Enhance COVID-19 Case-Fatality and Mortality Rates in the United States. Innovation (N Y). 2020 Nov 25;1(3):100047.

29. Pacheco H, Díaz-López S, Jarre E, Pacheco H, Méndez W, Zamora-Ledezma E. NO2 levels after the COVID-19 lockdown in Ecuador: A trade-off between environment and human health. Urban Clim. 2020 Dec;34:100674.

30. Raciti L, Calabrò RS. Can volcanic trace elements facilitate Covid-19 diffusion? A hypothesis stemming from the Mount Etna area, Sicily. Med Hypotheses. 2020 Jun 27;144:110058.

31. Saha J, Chouhan P. Indoor air pollution (IAP) and pre-existing morbidities among under-5 children in India: are risk factors of coronavirus disease (COVID-19)? Environ Pollut. 2020 Jul 15;266(Pt 2):115250.

32. Cole MA, Ozgen C, Strobl E. Air Pollution Exposure and Covid-19 in Dutch Municipalities. Environ Resour Econ (Dordr). 2020 Aug 4;1–30.

33. Wu X, Nethery RC, Sabath BM, Braun D, Dominici F. Exposure to air pollution and COVID-19 mortality in the United States: A nationwide cross-sectional study. medRxiv. 2020 Apr 7;

34. Wu X, Nethery RC, Sabath MB, Braun D, Dominici F. Air pollution and COVID-19 mortality in the United States: Strengths and limitations of an ecological regression analysis. Sci Adv. 2020 Nov;6(45).

35. Wang Y, Di Q. Modifiable areal unit problem and environmental factors of COVID-19 outbreak. Sci Total Environ. 2020 Oct 20;740:139984.

36. Yao Y, Pan J, Wang W, Liu Z, Kan H, Qiu Y, et al. Association of particulate matter pollution and case fatality rate of COVID-19 in 49 Chinese cities. Sci Total Environ. 2020 Nov 1;741:140396.

37. Zhu Y, Xie J, Huang F, Cao L. Association between short-term exposure to air pollution and COVID-19 infection: Evidence from China. Sci Total Environ. 2020 Jul 20;727:138704.

38. Zoran MA, Savastru RS, Savastru DM, Tautan MN. Assessing the relationship between ground levels of ozone (O3) and nitrogen dioxide (NO2) with coronavirus (COVID-19) in Milan, Italy. Sci Total Environ. 2020 Oct 20;740:140005.

39. Borro M, Di Girolamo P, Gentile G, De Luca O, Preissner R, Marcolongo A, et al. Evidence-Based Considerations Exploring Relations between SARS-CoV-2 Pandemic and Air Pollution: Involvement of PM2.5-Mediated Up-Regulation of the Viral Receptor ACE-2. Int J Environ Res Public Health. 2020 02;17(15).

40. Vasquez-Apestegui V, Parras-Garrido E, Tapia V, Paz-Aparicio VM, Rojas JP, Sánchez-Ccoyllo OR, et al. Association Between Air Pollution in Lima and the High Incidence of COVID-19: Findings from a Post Hoc Analysis. Res Sq. 2020 Jul 6;

41. Rodriguez-Diaz CE, Guilamo-Ramos V, Mena L, Hall E, Honermann B, Crowley JS, et al. Risk for COVID-19 infection and death among Latinos in the United States: Examining heterogeneity in transmission dynamics. Ann Epidemiol. 2020 Jul 22;

42. Zhu Y, Xie J, Huang F, Cao L. Association between short-term exposure to air pollution and COVID-19 infection: Evidence from China. Sci Total Environ. 2020 Jul 20;727:138704.
